# Virtual Health Research Capacity Strengthening in Low- and Middle‑Income Countries: A Systematic Integrative Review

**DOI:** 10.5334/aogh.4543

**Published:** 2025-03-11

**Authors:** Chelsea M. McGuire, Nikolina Boskovic, Bolatito Betty Fatusin, Pius Ameh, Taylor Reed, Priyanka Jethwani, David Flynn, Jo Cooke, Robert Saper

**Affiliations:** 1 Family Medicine Specialty Training Program, Lesotho‑Boston Health Alliance, Leribe, Lesotho; 2 Department of Family Medicine, Boston University Chobanian & Avedisian School of Medicine, Boston, USA; 3 Curta, Seattle, Washington, USA; 4 Family Medicine Department, Federal Medical Centre, Abeokuta, Nigeria; 5 Department of Family Medicine, Federal Medical Centre, Keffi, Nigeria; 6 Department of Family Medicine, Engela District Hospital, Helao Nafidi, Ohangwena Region, Namibia; 7 Department of Biology, Boston University, Boston, USA; 8 Department of Family and Community Medicine, University of Texas Southwestern, Dallas, Texas, USA; 9 Boston University Chobanian & Avedisian School of Medicine, Massachusetts, USA; 10 School of Allied Health Professions, Nursing and Midwifery, The University of Sheffield, Sheffield, South Yorkshire, England; 11 Nancy J. And Michael F. Roizen Chair of Wellness, Department of Wellness and Preventive Medicine, Cleveland Clinic, Cleveland, USA

**Keywords:** health research, capacity strengthening, online learning, clinicians, LMICs, integrative review, health equity

## Abstract

*Background:* Effective and scalable strategies are needed to develop health research capacity in low- and middle‑income countries (LMICs). Health research capacity strengthening (HRCS) focuses on boosting production and utilization of health research, with clinicians as key target participants. Despite the increased prevalence of virtual HRCS programs, there has been no review of the evidence for those targeting LMIC clinicians to date.

*Objective:* This review characterizes the use of virtual tools in HRCS programs for clinicians in LMICs and describes the impacts, facilitators, and barriers associated with these programs.

*Methods:* Following our protocol (PROSPERO; CRD42020152510), we employed an integrative review methodology. We adapted Cooke’s Research Capacity Development for Impact framework by adding “equity” as a new domain and used it to evaluate programmatic impacts. We retrieved relevant articles from five databases and gray literature. Included articles were extracted and stratified by degree of virtual delivery. We analyzed virtual tool usage via content analysis. Using NVivo, we coded until theoretical saturation and analyzed data using the constant comparison method.

*Findings:* From 1397 articles, 58 met inclusion criteria. Most programs were hybrid, and e‑courses were the most used virtual tool. Articles described impacts across all framework domains; the most discussed were skills and confidence building. Facilitators included user‑friendly platforms, interactive content, and strategies to improve program access, including financial and technological support. Some programs incorporated hybrid strategies to foster trust among participants and virtual mentors. Barriers included a lack of or an unfavorable local research culture.

*Conclusions:* Recommendations from this review may guide the design and implementation of virtual HRCS programs for LMIC clinicians. These include selecting well‑fitted program participants, intentionally designing program structure and content, conducting needs assessments or pilots, incorporating equity as a programmatic target, ensuring longitudinal program evaluation and monitoring, and utilizing a comprehensive conceptualization of program sustainability.

## Introduction

Health research is widely recognized as a global public good with potential to improve health systems, policy, practice, and equity [1]. Health research capacity strengthening (HRCS) is the “process of empowering individuals, institutions, organizations and nations to: define and prioritize problems systematically; develop and [use research methods to] scientifically evaluate [causes and] appropriate solutions; and share and apply the knowledge generated” [2]. It is conducted via projects ranging from small‑scale training programs targeting individuals to systematic and multi‑level interventions [3]. Despite significant HRCS efforts in low‑ and middle‑income countries (LMICs) over the past 25 years, gaps remain in the capacity needed to harness the power of health research to advance LMIC population health and development [4, 5].

Nurses [6, 7], physicians [8, 9], and allied health professionals [10, 11] are key targets for HRCS efforts globally. Clinician‑researchers have been described as “bridgers,” increasing the generation of clinically relevant research and direct dissemination of new knowledge and practices [12]. Clinicians’ participation in all types of health research helps ground investigations in the realities of clinical practice. This research is ideally positioned to advance healthcare delivery, impact social determinants, and improve population health [8, 13].

Barriers faced by HRCS efforts in LMICs include inequitable access to training opportunities [5, 14], prohibitive costs, lack of innovative finance mechanisms [2], post‑program “brain drain” [15], unsustainability, and lack of high‑quality evaluations [4]. In response, there are calls for increased use of virtual tools to support HRCS programs [5, 8, 16]. Reported benefits of online HRCS include disseminating standard curricula widely to participants in their home countries, increased and sustained access to field experts via e‑mentoring, and potential automation of monitoring and evaluation [17, 18]. There has been a steady movement of medical and research education to virtual platforms over the past two decades [18–20], a trend established well before coronavirus disease 2019 (COVID‑19) pushed its acceleration. Yet, the ability of virtual HRCS to capacitate clinician‑researchers in LMICs, as well as facilitators and barriers to these programs remains poorly understood.

### Authors’ context

We undertook this systematic review to address this literature gap and inform development of the AfriWon Research Collaborative, an online research training and mentorship program for family physicians in sub‑Saharan Africa. The program, piloted in 2019 by CM, PA, and BF, is detailed elsewhere [21], will benefit from insights gained in this review for future program cycles. The primary review team (CM, NB, and TR) are based in the United States (U.S.), and collaborators (PA and BF) are based in Nigeria. JC is the author of the Research Capacity Development for Impact framework [13, 22].

## Methods

Using the Whittemore and Knafl stages of integrative reviews, which allow for the inclusion of diverse methodologies and avoid restriction to only experimental studies [23], we sought to comprehensively examine all relevant literature. We registered the protocol with the International Prospective Register of Systematic Reviews, (PROSPERO CRD42020152510) initially in September 2019 and made two documented amendments [24]. The protocol followed the Preferred Reporting Items for Systematic Reviews and Meta‑Analysis (PRISMA) guidelines [25].

### Problem identification

A preliminary search identified prior reviews covering HRCS programs in LMICs [4], non‑academic HRCS models in sub‑Saharan Africa [26], and mentorship programs designed for health practitioners in rural and remote contexts [27]. Other reviews also highlighted e‑learning for health professionals, and some specifically touched on medical e‑learning in LMIC contexts [20]. However, no existing reviews were specific to virtual HRCS programs targeting LMIC clinicians. From this preliminary search we defined our research questions: (1) How are virtual tools used in HRCS programs for clinicians in LMICs? (2) What are the impacts of virtual HRCS programs for clinicians in LMICs? (3) What are the facilitators and barriers to virtual HRCS programs for clinicians in LMICs? To answer these questions, we defined specific inclusion and exclusion criteria (Box 1).

**Box 1. Inclusion and Exclusion Criteria**

**Table:** 

Population	Inclusion	Clinicians, defined as individuals or groups of health professionals trained in the delivery of clinical care to patients (e.g., physicians, nurses, psychologists, clinical pharmacists, and post‑graduate trainee clinicians) Low‑ and middle‑income countries as defined by the World Bank
	Exclusion	Undergraduate health profession students (e.g., medical or nursing students)
Intervention	Inclusion	Health research capacity strengthening (HRCS) activities that include efforts to increase the ability of targeted individuals to conduct or utilize health research May include any type of HRCS training, instruction, or education program regardless of program length; may be academic or non‑academic Entirely or partially delivered by virtual tools, defined as any program activity (such as a webinar, discussion forum, social media discussion, or e‑mentorship) that is carried out, accessed, or stored by means of a computer or mobile phone connected to the internet
Comparators	Inclusion	May have a comparison group as part of evaluation, but not required
Outcomes	Inclusion	Must report program delivery outcomes; however, no specific metrics required
Study Design	Inclusion	All evaluation designs (including qualitative studies, randomized controlled trials, quantitative non‑randomized, quantitative descriptive, and mixed methods); *no exclusion based on lack of formal study design nor on degree of detail on data collection methods*
Other Criteria	Inclusion	All publication types (original research, brief reports, conference proceedings, gray literature project reports, and commentaries) Articles describing HRCS programs that were implemented prior to article publication and include a minimum of four of the following categories of information: 1) program objective, 2) program content/components, 3) program duration, 4) target population, and 5) funding source
	Exclusion	Non‑English language Articles published prior to January 1, 1990, given the World Wide Web became available that year [28] Unretrievable full text of article

**Figure 1:**
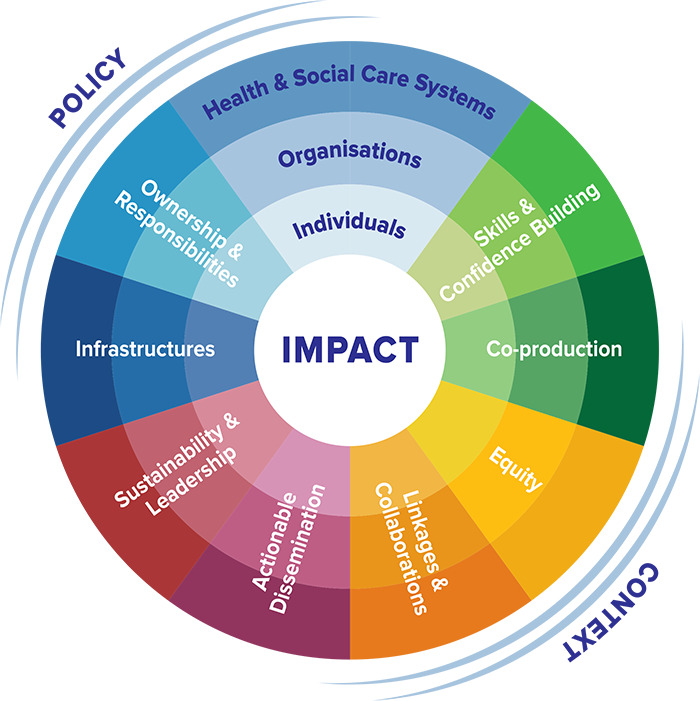
Modified Research Capacity Development for Impact framework [22]. Domain definitions are as follows: **skills and confidence building**—research capacity skills and confidence of individuals and groups, includes traditional research methodology and scientific writing skills, along with “boundary‑spanning skills,” such as relationship building; **co‑production**—research that is produced within the context where it will be useful and engages the “right” people in the research process; **equity**—reduction of health research capacity disparities between and within groups as a program output or outcome; may be related to program structures, such as the promotion of recruitment/retention of marginalized groups; **linkages and collaboration**—building connections among researchers and groups to co‑conduct research, including between diverse disciplines; **actionable dissemination**—sharing project results in ways that address the translational gap, communicate directly with target audiences, or call to action; **sustainability and leadership**—leadership development among participants and groups, especially leadership that calls for sustaining and expanding newly gained skills (e.g., undertaking new projects, participants of the program becoming leaders or mentors themselves, etc.), and program sustainability over time; **infrastructures**—development of structures to support research capacity including mentorship/supervision and project management structures, long‑term collaborative partnerships, research grant/funding arrangements, structures to help with governance and ethics, and/or organized information exchange events (e.g., virtual conferences); and **ownership and responsibility**—promoting individual or group engagement in research that is “owned” and transitioned to being a core component of the one’s work or organizational fabric (e.g., an organization specifically allocating time for clinicians to do research). The framework domains function across three structural levels (individuals, organizations, and health and social care systems) and are surrounded by two environmental factors (policy and context) that inform and are informed by each domain.

Our preliminary search led us to explore frameworks for analyzing research question 2. We identified six HRCS frameworks [13, 29–33] and systematically mapped the three most promising ones [22, 29, 30] against Cooke’s 2005 framework [13]. Through this process, we found that Cooke’s updated 2020 Research Capacity Development for Impact (RCDi) framework [22] was best suited for our analysis, given its nuanced, multilevel, and intersectional approach to evaluating HRCS program impacts. Cooke’s framework intentionally includes both process and outcome measures, which help to evaluate capacity strengthening progress more “sensitively and quickly” [13]. The framework was developed from HRCS experience in a high‑income context, and we noticed a gap in its ability to assess impacts on equity [34]. Consequently, we developed a modified RCDi framework (Figure 1), incorporating an additional domain denoting “equity” as a program output or outcome. We define the theoretical construct of “impact on equity” as the reduction in disparities in health research capacity between and within groups. Alongside equity, our framework encompasses the following domains: skills and confidence building (including traditional research capacity skills and boundary‑spanning relational skills); co‑production (research that is produced while working with end users of the research); linkages and collaboration (connections between researchers to co‑conduct research); actionable dissemination (sharing project results directly with target audiences to address the translational gap); sustainability and leadership (leadership development in ways that sustain or expand on programmatic gains over time); infrastructures (development of program management structures and long‑term collaborative partnerships); and ownership and responsibility (research being “owned” or seen as “core business” alongside clinical work) [22]. For detailed domain definitions, refer to the legend of Figure 1.

### Literature search

With a medical librarian (DF), we developed and refined search terms tailored to each database, encompassing “research capacity strengthening” and “LMIC.” The search, conducted in October 2019, spanned PubMed, Embase, Web of Science (Science and Social Science Citation Index), Education Resources Information Center (ERIC), and The Cochrane Library. Filters were applied to include articles published in English after January 1, 1990 (Supplement 1).

All identified articles underwent title/abstract screening using Rayyan [35]. Duplicate articles were removed. Two reviewers (CM and NB) reviewed all abstracts. Screening decisions were discussed, with conflicting decisions included to prioritize high sensitivity.

Articles potentially meeting inclusion criteria based on title/abstracts were exported to EndNote [36] for full‑text screening. Following agreement on three initially dual‑screened articles, NB and CM independently screened articles according to predefined population, intervention, comparison, outcomes, and study (PICOS) criteria (Box 1). Weekly discussions aided in resolving screening decisions. Authors were contacted for additional information when inclusion criteria were unclear.

During full‑text review, articles discussing future work or pending evaluations, as well as those citing other HRCS efforts, were flagged for further manual search. One reviewer (TR) conducted this supplementary search across published and gray literature sources, including PubMed, Boston University Library, and Google. Promising articles underwent screening by a second reviewer (NB or CM) prior to inclusion.

In March 2021, we repeated the same database searches to capture newly published articles. We screened abstracts, and promising full text articles were reviewed.

### Data evaluation

Data extraction: We extracted publication, program, and evaluation data from included articles. We concurrently assessed each article for the extent of virtual tool utilization in HRCS program delivery. Articles describing interventions where more than half of programming was delivered online were categorized as “high.” Those indicating significant, but less than half of, online delivery was defined as “moderate.” Articles describing a minor online adjunct alongside an otherwise in‑person program were defined as “minimal.”

Qualitative coding: We developed an *a priori* codebook using the modified RCDi framework domains, broad codes to capture facilitators and barriers, and codes for contextual details related to program and evaluation variables. CM and NB conducted independent open‑coding to refine codebook definitions, and the codebook was shared with other authors before finalization. All included articles were imported into NVivo [37] and independently coded by two reviewers, who then met to consensus code each article.

### Data analysis

We conducted descriptive analysis of program, evaluation, and publication characteristics. We examined which virtual tools were utilized by programs and what evaluation outcomes were reported through content analysis of extracted data. Each reviewer independently analyzed and categorized a subset of data, followed by collaborative meetings to establish final categories [38].

After categorizing articles based on virtual tool usage (high, moderate, or minimal), we initiated coding with articles classified as high usage, followed by moderate. We engaged in ongoing qualitative analysis using the constant comparison method [23]. Initially, one U.S.‑based author (CM, NB, or TR) was paired with a Nigeria‑based author (BF or PA) to independently review and categorize excerpts. Subsequent meetings facilitated consensus on categories. We continued coding iteratively, comparing excerpts with identified categories until theoretical saturation was achieved [39]. Categories were then synthesized into higher levels of abstraction using data display matrices, with input from all authors.

At the conclusion of the data analysis phase, BF, CM, NB, TR, PA, or PJ reviewed new articles identified in the second search and included full‑text articles for confirming information or additional insights. Articles containing new information were brought into the final analysis. Memoing was employed by reviewers throughout the integrative review to document emergent patterns and reflect on their role in data analysis [40].

To understand the methodological rigor of the studies in our sample, we used the Mixed Methods Appraisal Tool (MMAT) to assess program evaluations described in eligible empirical studies [41]. Articles that passed screening were independently assessed by two reviewers (NB, CM, and/or TR), with disagreements resolved through discussion. No articles were excluded from the study on the basis of MMAT results.

### Presentation

Data display and presentation were integral components of the analysis and interpretation processes. Final syntheses are presented via tables and figures.

## Results

### Characteristics of our sample

**Figure 2:**
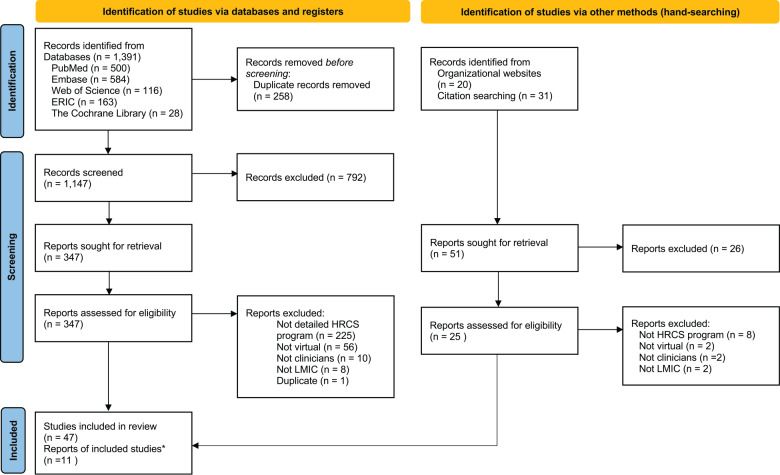
PRISMA flow diagram [25]. *Indicates number of publications identified via hand‑searching. HRCS, health research capacity strengthening; LMIC, low‑ and middle‑income country.

Our initial search identified 58 articles meeting inclusion criteria (Figure 2). Starting with those with high use of virtual tools, 25 were coded in NVivo, upon which saturation was met. No articles with minimal use of virtual tool use were coded. An additional 3 articles were identified in the second search, bringing the total to 28 articles informing the results. Citation information and characteristics of the remaining 39 articles are found in Supplement 2.

Key characteristics of the 28 articles are displayed in Table 1. Our analysis of articles’ publication, program, and evaluation characteristics are in Supplement 3. Notable characteristics of this sample include that program participants were commonly from sub‑Saharan Africa, followed by South Asia, and Latin America and the Caribbean. Participants ranged from five in one hybrid course, to over 600,000 in a fully virtual program. Most programs lasted 1–2 years. Many articles included no formal evaluation design. Articles reported a range of evaluation results. The most commonly reported metrics were the number of participants, their demographics, completion and attrition rates, and quantity of papers produced by participants. Some programs also reported changes in knowledge and rates of continued research involvement. Less frequently reported metrics included career advancement indicators, such as grants awarded, and individuals’ confidence in teaching the skills they had learned. Overall, 13 of 28 articles screened‑in for application of MMAT, and 7 of those 13 articles were deemed at low risk of bias (Supplement 4).

**Table 1:** Program characteristics (n = 28)^a^.

CITATION	HRCS PROGRAM OBJECTIVE(S) (PROGRAM SIZE)^b^	LMIC PARTICIPANTS’ COUNTRY OR REGION	CLINICIAN TYPE(S)	VIRTUAL COMPONENT(S) OF THE HRCS PROGRAM (FULLY VIRTUAL OR HYBRID)	EVALUATION DESIGN
Abawi 2016 [42]	To train healthcare professionals in sexual and reproductive health research (175)	59 countries in Africa, Asia, and North America	Midwives, nurses, and physicians	Online course, e‑mentorship, and online discussion group (fully virtual)	Non‑randomized
Aggarwal 2011 [9]	To compare impact and accessibility of online distance or on‑site training courses for scientists engaged in biomedical research (58)	India	Physicians	Online course (hybrid)	Randomized controlled trial
Atkins 2016 [43]^c^	To build postgraduate students’ health research capacity through online research work‑in‑progress meetings (NR)	India, Malawi, and Nicaragua	Junior researchers	Online course (fully virtual)	None
Barchi 2013 [44]	To train ethics committee members and researchers and evaluate the effectiveness of internet‑based learning (72)	Botswana	Ethics committee members	Online course and e‑mentorship (hybrid)	Randomized controlled trial
Bloomfield 2016 [45]	To enhance infrastructure and training necessary for early career researchers to conduct innovative and locally relevant non‑communicable disease research (563)	Argentina, Bangladesh, China, Guatemala, India, Kenya, Mexico, Peru, South Africa, and Tunisia	Junior researchers, and physicians	Online course, e‑mentorship, and online discussion group (hybrid)	None
Braunschweiger 2007 [18]	To train researchers on human subjects’ protection and responsible research conduct (>600,000)	26 countries in the Caribbean and South America	NR	Online course (fully virtual)	None
Byrnes 2019 [46]	To improve research capacity for non‑communicable diseases in Asian LMICs (48)	India, Malaysia, and Sri Lanka	NR	Online course and online repository (hybrid)	Mixed methods
CORDIS 2015 [47]^c^	To build capacity in health systems and services research in sub‑Saharan African universities (300)	South Africa and Uganda	Junior faculty	Online course, online discussion group, and online repository (hybrid)	Quantitative descriptive
Da Silva 2019 [48]^d^	To improve mental health research capacity of medical doctors, clinical nurses, and psychologists via collaborative hubs (5)	Argentina, Brazil, Ethiopia, Ghana, and Nepal	Clinical psychologists, nurses, and physicians	Online learning‑by‑doing and online discussion group (hybrid)	Qualitative
Decroo 2018 [49]	To improve operational research capacity of clinicians through a blended online and in‑person training program (6)	India and Kenya	Physicians and physiotherapists	Online course (hybrid)	Quantitative descriptive
Dodani 2012 [50]^e^	To equip physicians with epidemiology research skills (56)	Bangladesh and Pakistan	Physicians	Online course (hybrid)	Quantitative descriptive
Dodani 2008 [51]^e^	To equip physicians with epidemiology research skills (40)	Bangladesh and Pakistan	Physicians	Online course (hybrid)	Non‑randomized
Heller 2015 [52]^f^	To improve population health by building public health capacity via e‑learning at very low cost (117)	Eastern, Southern, and Western Africa, and Indian subcontinent	Clinicians	Online course, e‑mentorship, online discussion group, and online learning‑by‑doing (fully virtual)	None
Heller 2009 [53]^f^	To develop infrastructure, administrative capacity, and course content for online public health training (117)	23 countries in Africa, including Nigeria, Tanzania, and Uganda	Clinicians and physicians	Online course and online discussion group (fully virtual)	None
Loisel 2009 [54]	To enable trainees to contribute to the the ﬁeld of work of disability prevention with a transdisciplinary perspective (44)	Brazil, Canada, France, the Netherlands, and the United States	Clinicians	Online course (hybrid)	None
Mayor 2019 [55]	To build and strengthen health research capacities in Good Clinical and Laboratory Practice of trainees (18)	Liberia	Midwives, nurses, pharmacists, physicians, and physician assistants	Online course, e‑mentorship, and online discussion group (hybrid)	None
McGuire 2020 [16]^g^	To use peer mentorship to build health research capacity among family medicine trainees (8)	Lesotho	Clinicians	Online course, e‑mentorship, and online learning by‑doing (hybrid)	Mixed methods
Mill 2014 [56]	To enhance nurses’ qualitative research capacity and promote involvement in HIV policy via participatory action research (25)	Barbados, Jamaica, Kenya, South Africa, and Uganda	Nurses	Online course, e‑mentorship, and online learning‑by‑doing (hybrid)	None
Nazer 2021 [57]^g^	To increase research skills and productivity among clinicians with minimal research experience in the Middle East (11)	Egypt, Jordan, Oman, Saudi Arabia, and Sudan	Clinicians	Online course and e‑mentorship (hybrid)	None
Okewole 2020 [58]^g^	To train clinicians and junior mental health researchers in academic research skills (15)	Ethiopia, Malawi, South Africa, and Zimbabwe	Clinicians and junior researchers	Online course (hybrid)	Qualitative
Pilowsky 2016 [59]^e^	To develop opportunities and capacity for mental health research in LMICs (NR)	Afghanistan, Bangladesh, Brazil, Chile, Colombia, Ecuador, Ethiopia, Ghana, Guatemala, India, Kenya, Liberia, Malawi, Nepal, Nigeria, Pakistan, Peru, South Africa, Sri Lanka, Uganda, and Zimbabwe	Clinicians	Online course, e‑mentorship, online learning‑by‑doing, and online repository (hybrid)	None
Protsiv 2016 [60]^c^	To train health systems and services research doctoral students in conducting meta‑analytic studies (19)	South Africa and Uganda	Clinicians and pharmacologists	Online course and e‑mentorship (hybrid)	Mixed methods
Reynolds 2008 [61]^f^	To develop and pilot public health online diploma course for practitioners in developing countries (38)	Democratic Republic of Congo, Ghana, India, Nigeria, Pakistan, Sudan, and Turkey	Nurses and physicians	Online course and online discussion group (fully virtual)	Quantitative descriptive
Sharma 2017 [62]^e^	To conduct policy research to reduce the treatment gap for mental disorders in South Asia (52)	Afghanistan, Bangladesh, India, Nepal, Pakistan, and Sri Lanka	Psychiatrists and mental health researchers	Online course, e‑mentorship, online discussion group, and online repository (hybrid)	None
Silverman 2013 [63]	To improve institutional and individual research ethics capacity of Middle Eastern countries (NR)	Egypt, Jordan, Lebanon, Libya, Sudan, Syria, and Yemen	Ethics committee members	Online course, online discussion group, and online repository (hybrid)	None
Thakurdesai 2018 [64]	To develop academic and research competence for academic psychiatrists and postgraduate students (NR)	India	Psychiatrists	E‑mentorship, online discussion group, and online learning‑by‑doing (fully virtual)	None
Wright 2005 [6]^h^	To build research and leadership capacity of Latin American nurses in substance abuse and health promotion, with a specific focus on drug‑demand reduction (11)	Argentina, Brazil, Chile, Colombia, Ecuador, Mexico, and Peru	Nurses	E‑mentorship and online learning‑by‑doing (hybrid)	None
Wright 2015 [65]^h^	To build a cadre of academic health professionals with specialized knowledge and research skills in drug‑related issues (91)	Brazil, Colombia, Jamaica, Nicaragua, Chile, El Salvador, Ecuador, Peru, Costa Rica, Guatemala, Guyana, Mexico, Honduras, Panama, Trinidad and Tobago, Uruguay, Argentina, Bahamas, Barbados, Belize, Paraguay, and Suriname	Nurses, occupational therapists, physicians, pharmacists, and psychologists	E‑mentorship and online learning‑by‑doing (hybrid)	None

^a^Twenty‑eight articles used in the results section of the systematic review.

^b^Program size refers to the number of participants reported to be initially recruited.

^c^Publications on African Regional Capacity Development for Health Systems and Services Research (ARCADE) intervention.

^d^Publications on South Asian Hub for Advocacy, Research and Education on Mental Health (SHARE) hub interventions.

^e^Publications on same intervention (unnamed).

^f^Publications on the People’s Open Access Education Initiative (People’s‑uni) intervention.

^g^Publications included from the updated March 2021 literature search which contributed new information to the results.

^h^Publications on International Research Capacity‑Building Program for Health and Related Professionals to Study the Drug Phenomenon in Latin America and the Caribbean.

HIV, human immunodeficiency virus; HRCS, health research capacity strengthening; LMIC, low‑ and middle‑income country; NR, not reported.

### Virtual tools for HRCS

Seven articles in our sample described HRCS programs that were fully virtual, and the remaining were hybrid, combining in‑person and virtual delivery strategies. The virtual tools used by these programs fell into the following categories: online courses, online mentorship or coaching (e.g., “e‑mentorship”), online discussion groups or forums, online repository of resources, and online learning‑by‑doing. Online courses were the most used tool in our sample, followed by e‑mentorship.

### HRCS program impacts

Across the whole sample, we found some evidence of impact in all modified RCDi framework domains (Table 2). Impacts within the skills and confidence building domain and the sustainability and leadership domains were most frequently discussed in the articles. The least frequently discussed were impacts on equity, infrastructures, and co‑production. Substantial overlap across domains was noted and speaks to their interconnected nature. We explore these concepts below as we discuss major themes within each domain.

**Table 2:** Identified impacts of HRCS programs with illustrative quotes.

DOMAIN (ABBREVIATION)	IMPACT THEMES^a^	ILLUSTRATIVE QUOTES
**Skills and confidence building (SKILLS)**	Programs demonstrated various skill‑building impacts, with many reporting on traditional research skills, such as critical appraisal, data analysis, and research dissemination, while others also discussed gains in soft skills, including tolerance, interpersonal communication, and worklife balance (LINK/COLLAB)	2a) “The graduates are proven to have postgraduate level research and critical appraisal skills” [52] 2b) “I can say with great confidence that I have gained capacity not only in qualitative research but also in doing qualitative research across different countries and cultures” [56]
	Some programs specifically described impacts on participant confidence, which was tied to career advancement, taking a stand on issues, and building partnerships (SUS/LEAD)	2c) “I currently coordinate operational research activities with a lot of confidence and competency as an alumni of Peoples‑uni” [52] 2d) “Based on the program and the experience of realizing the research project, I[’ve had] growing confidence to take a stand about the problem in various professional contexts” [6]
**Co‑production (COPROD)**	Virtual networks allowed researchers to work together on projects that were relevant to their own communities and local clinical contexts (LINK/COLLAB)	2e) “During a discussion on instruments to measure stress, the Presumptive Stressful Life Events Scale was considered (Singh et al., 1984), [however] (...) it was realized that the scale requires revision to make it more relevant [to the network of researchers’ current cultural context]. Members of eJCIndia (...) working in academic institutions in different parts of the country volunteered to be a part of [this] multicenter project” [64] 2f) “The study (...) involved a survey of the value of the use of information technology in the implementation of guidelines to improve clinical or public health practice. None of the participants had previously performed research together, and 44 of the possible 48 members of the [virtual] Alumni group at the time participated in the data collection” [52]
	Some programs intentionally involved key decision‑makers and institutions in the research process (LINK/COLLAB, SUS)	2g) “To build a sustainable model of research capacity building in a region with inadequate and inequitable resources, it is imperative to involve (...) regional institutes with complementary roles and expertise. (...) Some of SHARE [South Asian Hub for Advocacy, Research and Education on Mental Health] partner institutes (...) work closely with the Ministry of Health in their respective countries. This gave an opportunity to the SHARE core team to initiate a dialog with the government agencies and decision makers and conduct training programs and dissemination programs for the relevant government bodies” [62] 2h) “The use of a PAR [participatory action research] design also incorporated the opportunity for ongoing, deliberative collaboration between nurses and decision‑makers” [56]
**Equity (EQ)**	Some projects were explicitly designed and implemented to address systemic and individual barriers to access and participate in research, including collaborative agreements to promote power‑sharing, targeted funding or selection of participants, using flexible e‑learning strategies to reach those who may be otherwise excluded, and providing resources specifically needed in LMIC contexts (CO‑PROD, INF, SKILLS)	2i) “Individuals eligible for support by MERETI [Middle East Research Ethics Training Initiative] include those from the World Bank‑designated LMIC categories. While individuals from HICs in the region are not eligible for funding from the program, they may participate as self‑funded students” [63] 2j) “While efforts to redress the gender imbalance at SSA institution continues, providing opportunities through e‑Learning, addressing some of the barriers that may impact the training of women, is an important achievement of ARCADE HSSR: Of a sample of 20 students (...) half had children under the age of 5 years. (...) childcare responsibilities may prevent parents from taking part in research. Particularly, travel (...) is often out of the question. Through (…) blended learning ARCADE HSSR has provided excellent courses for caregivers of children, policymakers, and others who are unable to travel distances to partake in cutting edge training” [47]
	Equity was not explicitly addressed or measured in most articles and one program specifically noted an inability to impact equity (CO‑PROD, INF, SKILLS)	2k) “Despite the best efforts to provide equitable research capacity‑building opportunities (...) [they] remained skewed to settings where the infrastructure already existed” [62]
**Linkages and collaboration (LINK/COLLAB)**	Collaboration between researchers with diverse experiences can enhance the quality of HRCS programs and research outputs (DISS, COPROD)	2l) “The (...) program applied team science principles by bringing together a group of investigators with diverse disciplines, management styles, local and regional global health needs, core competencies, and training program objectives working towards a common goal. By working together and leveraging on their training expertise, COE [Collaborating Centers of Excellence] training representatives closed gaps in their training programs (...) and collaborated on proposals, securing additional funding (...) to enhance their respective training programs” [45] 2m) “The solutions offered in eJCIndia group discussions lead to an improvement in the quality of studies designed and papers written by members (...) [via] knowledge pooling by members who have expertise in diﬀerent ﬁelds of psychiatry and psychiatric research” [64]
	Some programs encouraged collaboration via team mentorship that linked researchers at various career stages (SUS/LEAD)	2n) “The SHARE capacity‑building team realized that the model based on one–one mentoring by senior researchers may not be sustainable (...) in a region where there are few trained researchers; therefore, the SHARE team endeavored to form a network of mentors at different career stages. Thus, at the end of its 5 years, SHARE has successfully brought together a bank of researchers with varied experience and areas of expertise, to provide guidance or career advice to young or early career researchers” [62] 2o) “This programme built linkages between senior researchers, junior researchers, and students. Faculty from MU [Makerere University], SU [Stellenbosch University], MA [Malawi University College of Medicine], and MUHAS [Muhimbili University of Health and Allied Sciences] attended meetings during which students worked on research protocols, thus also creating research linkages between the staff. The meetings clarified the staff’s research interests, and linked students to staff, both within institutions and between institutions. Thus, many senior researchers could assist junior researchers and students in developing their research proposals” [47]
	The formation of virtual research networks, including those which extend beyond initial program targets, were highlighted as important; however, the impact of such networks can be difficult to quantify as they may occur over long periods of time (SUS/LEAD)	2p) “Formation of peer network of researchers was the pivot of success of the SHARE program” [62] 2q) “There were some research programmes on the periphery of the consortium that emerged during the project and represented collaboration between the two hubs. (...) No results are available yet from these studies” [47]
**Actionable dissemination (DISS)**	In addition to the commonly reported metric of number of research publications by participants, some programs also reported other forms of dissemination, such as conference submissions and presentations (LEAD/SUS)	2r) “Activities have led to the preparation of about 20 articles in three years” [64] 2s) “A total of 154 research presentations were given by trainees at national and international conferences” [64]
	Examples of actionable dissemination included directly sharing research findings with policymakers and stakeholders, and the use of non‑traditional formats for dissemination of results, such as policy briefs	2t) “Trainees have also had the opportunity to exchange their research findings with senior policymakers and practitioners” [46] 2u) “In order to highlight the rich, in‑depth qualitative findings, we shared findings using a wide range of non‑traditional formats for a variety of target audiences. These included lay summaries, policy briefs, and concise recommendations for decision‑makers and front‑line nurses” [56]
	Some HRCS programs led to trainees participating in further research, taking leadership roles in their countries, expressing interest in, or pursuing higher research degrees, and advancing their careers (COPROD, INF, DISS)	2v) “More than 50% of ASCEND [Asian Collaboration for Excellence in Non‑Communicable Disease] trainees commenced higher research degrees during or following their participation in the ASCEND program. These included PhD, Masters, or MD programs (...) with mentoring and supervisory support provided by the international ASCEND faculty” [46] 2w) “I substantially improved my abilities for research and now I work on advanced nursing research projects with greater social impact, with my graduate students and professors. (...) I hold a leading position in the Faculty, where scientific knowledge is generated, applied, and spread; (...) services are extended to society; and management is realized, which means that ways of financing projects are looked for” [6]
**Sustainability and leadership (SUS/LEAD)**	The “training of trainers” model, wherein alumni or sometimes participants still within the program go on to train others in research, can support program sustainability (LINK/COLLAB)	2x) “A number of the alumni are cascading their knowledge to others through their own local teaching and are applying for research grants themselves and with colleagues” [52] 2y) “Team members described their increased confidence in qualitative and participatory research methodologies, with many members taking on teaching and mentoring roles in their own institutions by the end of the project, a clear indicator of the sustainability of the research capacity that has developed” [56]
	Funding and resource availability are essential for continued sustainability at all levels, with the organizational level being the most challenging to achieve	2z) “Sustainability of the program, ASCEND was largely based on financial viability and external funding. A sense of ownership by local institutions involved in the program would be critical for sustainability (...), however, without continual funding and further strengthening of capacity of these institutions, this is potentially challenging. At [an] individual level, the benefits of the research capacity strengthening program can be maintained, adapted, and developed. But sustainability of research capacity at [the] organizational level requires system and organizational level interventions” [46]
	Strategies to promote sustainability included encouraging local ownership, use of virtual networks for networking connections, ensuring access to resources beyond program‑end, and actively creating regional conditions that favor long‑term sustainability (INF, OWN, LINK/COLLAB)	2aa) “A momentous success of SHARE has been the achievement of sustainability and accessibility of training opportunities in the region (...) by developing various (...) virtual platforms for short courses, and by offering ‘classic’ lectures on mental health in open‑source formats for the global community. (...) The strength of the course was the cascade model, thereby ensuring that master trainers are trained in the region to ensure the momentum of further training” [62] 2bb) “Due to the hierarchical structure of academia in the Middle East region, junior trainees reported difficulties assuming major roles on their institution’s research ethics committee and implementing research ethics into the curriculum. To alleviate these difficulties, prior to trainee selection, we discuss with top university officials how they will promote and support trainee efforts when they return home, as well as selecting senior and junior faculty from the same institution so that junior trainees could be supported in advocating for and implementing research ethics activities” [63]
**Infrastructures (INF)**	Infrastructure impacts included formation of research ethics governance structures, research training agreements between universities, and the production of research training manuals	2cc) “Double Ph.D. degree training agreements were signed between SU [Stellenbosch University], MU [Makerere University] and SU and KI [Karolinska Institutet]” [47] 2dd) “An NVivo 8 resource manual was created by a Canadian RA [research assistant] and distributed to teams” [56]
	A few programs developed guidelines for collaboration, including ones specific to authorship, as a means of promoting equity in publication (EQ)	2ee) “We attempted to strategically design small research teams to carry out the research and to work together on the publication of findings. The Principles for Research Collaboration (...) clearly outlined the authorship guidelines and helped to ensure that LMIC team members had the same opportunities to participate in publications as the Canadian team members did” [56] 2ff) “For the process of publication, another letter of agreement between CICAD [Comisión Interamericana para el Control del Abuso de Drogas] and University of Alberta‑Faculty of Nursing was signed, detailing the nature and authorship of the manuscripts that would result from this unique experience” [65]
**Ownership and responsibility (OWN)**	Some HRCS programs precipitated ownership and responsibility by prioritizing and developing structures for local participation of diverse actors, from junior researchers to decision‑makers (CO‑PROD)	2gg) “The ability of more junior team members to mentor others provided evidence that the qualitative capacity was not only locally‑owned, but also sustainable following completion of the project” [56] 2hh) “The involvement of the leadership hubs, including front‑line practitioners, managers, and decision‑makers, reinforced the importance of qualitative research and helped to ensure that the capacity building was locally‑owned” [56]

^a^Most themes were noted during the analysis to overlap with other domains, these overlaps are denoted by noting the abbreviation of the additional domain(s) in parentheses.

HIC, high‑income country; HRCS, health research capacity strengthening; LMIC, low‑ and middle‑income country.

#### Skills and confidence building

HRCS programs demonstrated a variety of skill‑building impacts. Descriptions of participants’ improvement in traditional research skills, such as qualitative research, data collection and analysis, and research dissemination, were common (Quote 2a) [52]. Others discussed gains in “soft skills,” including interpersonal communication, tolerance, and worklife balance (Quote 2b) [56].

The studies discussing participants’ gains in soft skills overlapped with the RCDi domain of linkages and collaboration, as these skills improve one’s ability to co‑conduct research. Other programs described improvements in participant confidence, in building partnerships, taking a stand on issues, and advancing their careers. This latter theme had significant overlap with the RCDi domain of sustainability and leadership (Quotes 2c–d) [6, 52].

#### Co‑production

Both themes that emerged under this domain overlapped with the linkages and collaboration RCDi domain. Virtual networks allowed researchers to work together on projects that were relevant to their own communities and local clinical contexts (Quotes 2e‑f) [52, 64].

Several programs guided participants to involve key decision‑makers and institutions in their research processes. This also impacted sustainability and leadership since the experience engaging with these stakeholders in research production grew participants’ leadership skills, afforded opportunities for research dissemination, and led to further collaborations (Quotes 2g–h) [56, 62].

#### Equity

Some projects were explicitly designed and implemented to impact equity by addressing systemic and individual barriers to participation in research. Examples of this included collaborative agreements to promote power‑sharing, targeted funding, or selection of participants (Quote 2i) [63], using flexible e‑learning strategies to reach those who may be otherwise excluded (Quote j) [47] and providing resources specifically needed in LMIC contexts. Mill et al. discussed their intention to decolonize their HRCS efforts, by obtaining “ethical approval to store the raw data in each of the study countries” and promoting shared leadership via “three co‑principal investigators” [56]. Building project management and governance structures in this way allowed implementers to impact equity via the RCDi infrastructures domain, which was otherwise rare in our sample. Equity was not discussed or measured in most articles in our sample, and one program noted a specific inability to positively impact equity (Quote 2k) [61].

#### Linkages and collaboration

Some studies reported that collaboration among researchers with diverse experiences improved both HRCS program quality and programmatic research outputs, including higher‑quality study designs, research papers, and increased success in securing funding (Quotes 2l–m) [45, 64]. This also aligns with the RCDi domains of co‑production and dissemination, as these collaborations often include local researchers who can help translate research outputs into action in their community. Some programs fostered collaboration by using team mentorship, which enhanced the connectedness of researchers at various career stages (Quotes 2n–o) [47, 62] and, in turn, supported leadership development and program sustainability. The formation of research networks was also highlighted by some articles as important and as a driver of sustainability, though quantifying their impact poses challenges (Quotes 2p–q) [47, 62].

#### Actionable dissemination

In addition to the commonly reported metric of research publications by participants (Quote 2r) [64], some articles reported other dissemination methods, including conference submissions and presentations (Quote 2s) [64]. These activities foster leadership skills, with conference presentations affording more direct opportunities for target audience communication. However, in one program, authors noted that “the number of publications after the workshop was not signiﬁcant” [50]. Other actionable dissemination methods included directly sharing research findings with policymakers and stakeholders, as well as using non‑traditional formats, such as policy briefs (Quotes 2t–u) [46, 56].

#### Sustainability and leadership

Impacts included participation in further research, assumption of leadership roles, the pursuit of higher research degrees, and other career advancement outcomes (Quotes 2v–w) [6, 46]. Trainees’ post‑program experiences included outcomes that overlapped with actionable dissemination, as trainees went on to share and apply their research, and with infrastructure, as they went on to earn research grants or build new research partnerships.

The “train the trainers” or cascade model, wherein alumni or participants in the program go on to train others in research, was a sustainability strategy that was highlighted by a few articles in our sample (Quotes 2x–y) [52, 56]. Another common theme was that funding and resource availability are essential for continued sustainability at all levels of the RCDi framework, with sustainability at the organizational level being the most challenging to achieve (Quote 2z) [46].

Strategies to promote sustainability were diverse and included encouraging local ownership, use of virtual networks for establishing professional connections, ensuring access to resources beyond a program’s end, and actively creating regional conditions favoring long‑term sustainability (Quotes 2aa–bb) [62, 63].

#### Infrastructures

Some infrastructure impacts were described, including establishment of research ethics governance structures, university research training agreements (Quote 2cc) [47], and production of research training manuals (Quote 2dd) [56]. A few highlighted strategies at the intersection of infrastructure and equity, such as creating and utilizing multinational research collaboration and authorship guidelines to help promote equitable distribution of research benefits (Quotes 2ee–ff) [56, 65].

#### Ownership and responsibility

Examples of ownership were limited in our sample. One article described achieving impacts within the ownership and responsibility domain by prioritizing and developing structures for local participation of diverse LMIC actors, ranging from junior researchers to front‑line practitioners and decision‑makers (Quotes 2gg–hh) [56]. Engaging the “right” people in the research process, e.g., co‑producing research, can serve to reinforce local ownership and support HRCS sustainability. Mayor et al. reported that their program did not lead to local ownership owing to it being funded by “the global North” and lacking “South‑South collaborations”; they call for others to prioritize “locally led and run initiatives that draw on existing regional capacities and funds to ensure local ownership” [55].

### HRCS program facilitators and barriers

**Figure 3:**
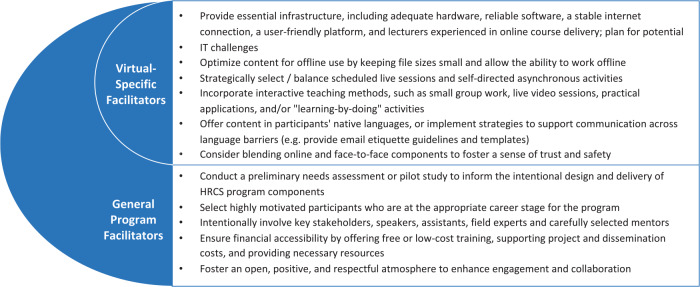
HRCS program facilitators. Summary of identified virtual‑specific and general HRCS program facilitators.

We grouped the identified facilitators and barriers first by those specific to virtual programs and then those relevant to any HRCS program, virtual or not. Key facilitators are summarized in Figure 3. Themes that showed up as facilitators when present and barriers when absent are presented as facilitators. Illustrative quotes to support the findings can be found in Supplement 5.

#### Virtual‑specific facilitators and barriers

##### E‑platform selection and use

To facilitate online course development, some programs selected familiar or user‑friendly platforms, had back‑up plans for IT failure, and ensured that lecturers had prior experience in online course delivery. In some cases, non‑user‑friendly platforms and those with security access limitations posed a barrier to accessing online learning.

##### Internet access

Some articles identified improving internet access within LMICs as a facilitator to e‑learning. However, a variety of creative strategies, such as keeping file sizes small or allowing participants to work offline until internet access was available, were necessary elsewhere to facilitate online participation. Some ensured participants had adequate hardware, software, and/or internet connection before the program started. In other programs where this was not done, poor quality of internet connection or virtual interfacing equipment limited or prevented participation.

##### Delivery and design strategies

While some programs highlighted the benefits of scheduled, synchronous sessions, others elected for flexible, self‑directed, and asynchronous approaches. In programs with synchronous components, the differences in time zones sometimes presented a challenge for participants. Interactive sessions via small group work or live video sessions, the use of practical application, and “learning‑by‑doing” were described as facilitators in several programs. According to Abawi et al., “interactivity [is] one of the key components that makes the online approach to capacity building effective” [42]. Not allowing students to use real‑world problems was named as a barrier to learning in one program. However, in some instances, the time‑intensiveness of interactive course elements became a barrier to participation for both learners and faculty.

##### Language considerations

Programs used creative strategies to be responsive to language preferences, norms, and skill levels. While some programs provided content in participants’ native languages, others outlined specific strategies to facilitate online communication despite language differences, such as minimizing acronym use and providing email etiquette guidelines and templates. Other programs noted language differences as a barrier to recruitment or active participation, especially within programs implemented in multiple countries.

##### Hybrid approach

Some hybrid programs noted that, by combining face‑to‑face and online components, one can build a sense of trust and safety. One program specifically recommended using a combination of in‑country and distance e‑mentorship to facilitate overall program effectiveness.

##### Characteristics of virtual HRCS

The ease of adapting, ease of evaluating, ease of monitoring, and ease of sharing online HRCS content were described as facilitators. However, some noted challenges in evaluating online programs, such as the difficulty measuring the degree to which participants were learning or benefiting from online content. Another program noted that participants lost access to course resources and networks after the programs ended. Although low recurring costs of virtual HRCS programs were identified as potentially supporting cost‑effectiveness and scalability, few articles reported programmatic cost details to support this claim.

#### General facilitators and barriers

##### Design informed by needs assessment

Starting with a needs assessment can focus program efforts and bring intentionality to the design and delivery of HRCS program components, especially with the different design considerations for online versus in‑person courses. A few articles in our sample reported issues with poor design, structure, and/or course content resulting in barriers to program implementation or effectiveness.

##### Participant characteristics

Selection of participants who are highly motivated and at the right point in their research career was discussed as a facilitator. Other programs noted a lack of uniform prerequisite skills among trainees as a barrier.

##### Program personnel characteristics

HRCS programs were facilitated by intentional involvement of specific stakeholders, speakers, and field experts, including statisticians and medical writers. Additionally, careful selection of mentors and peer mentors and the involvement of research assistants and graduates were named as facilitators. Some programs noted barriers arising from the challenge of matching the availability and/or appropriate expertise of mentors and mentees.

##### Program accessibility

Programs facilitated participation using various strategies, including making training free, supporting research and dissemination costs, and ensuring participants have the necessary ability, technology, and physical resources to successfully attend programs.

##### Research environment

Cultivation of an open, positive, and respectful atmosphere with the program is an important facilitator. A challenging external climate, such as political tensions, unfavorable administrative regulations, lack of research culture or infrastructure, or in some instances, the stigmatization or undervaluing of the research topic or methods, can pose a barrier to attempts at HRCS. Mathai et al. reported that stigma associated with mental health was a “significant barrier to mental health research in Kenya” [67].

## Discussion

Most identified HRCS programs targeting clinicians in LMICs were hybrid instead of fully virtual. Programs used a variety of tools, including online courses, e‑mentorship, online discussion forums, online repository of resources, and online learning‑by‑doing. Across our sample, we found examples of impact within all domains of the modified RCDi framework [22], including equity (Figure 1). Skills, confidence building, and sustainability were the most discussed impacts. We found examples of virtual networks permitting LMIC researchers to remain in their home communities to conduct research locally, enhancing contextual relevance. Team mentorship and “training of trainers” models were highlighted in some articles as strategies for developing individual leadership and program sustainability at the organization and systems levels, albeit with challenges in measuring higher structural‑level impacts. Rarely, articles acknowledged that their HRCS programs were unable to significantly improve equity or build ownership. Facilitators included user‑friendly platforms, interactive content, and intentional selection of both participants and program personnel, while barriers included a lack of or unfavorable local research culture.

The prevalence of hybrid programs in our sample may signal a transition from in‑person to online formats or represent intentionally designing programs that first build trust via an in‑person component before moving online, as was highlighted by Usher et al. [66]. A number of programs in our sample utilized virtual mentorship, which can ease time and distance constraints, especially in multi‑country programs. The flexibility of e‑mentorship can help address a key barrier to HRCS in LMIC contexts—the overall shortage of skilled research mentors—and could help unlock the numerous benefits of high‑quality mentorship for individual researchers, institutions, and healthcare systems [68]. Several programs in our sample utilized some version of the “training of trainers” model, which is often touted as a cost‑efficient strategy for capacity strengthening. However, in practice, the model’s effectiveness can be limited by insufficient investments in time, training, and resources and thus must be designed and evaluated carefully [69].

Our review introduced and applied a modified RCDi framework [22], which considers HRCS across individual, organization, and systemic levels. We observed extensive interconnectedness among all framework domains, particularly regarding equity. Such interconnectedness may well be synergistic. We included equity within the framework to highlight the importance of this as an underlying principle for HRCS in LMICs. Equity may be considered a lens through which to examine all impacts, since HRCS aims to bridge capacity gaps for locally led research and advance health equity [70]. Nevertheless, we recommend maintaining equity as a distinct framework domain to ensure its explicit consideration in program design and evaluation.

### Recommendations

On the basis of our findings, we recommend the following strategies to those interested in designing, implementing, and/or evaluating virtual HRCS programs, especially in LMIC contexts.

1. Select well‑fitted participants for the program you are building.
  Successful participation in a virtual HRCS program requires self‑motivated individuals at the appropriate career stage who have adequate time, technology, and network access. Intrinsic motivation enhances virtual learning success [71]. Aim for participants interested in future leadership and committed to teaching or mentoring, ensuring a path to sustainability and ownership. “Train the trainer” programs should consider factors necessary for sustainability at all operational levels, as per the 2018 TRAIN framework [69]. However, exercise caution to avoid creating barriers to equity, such as screening criteria based on degree of internet access, which could exclude disadvantaged participants. Collaborating with local partners can help identify well‑suited participants and develop proactive measures to address equity, including resource provision for those with limited virtual access.
2. Intentionally design program structure and content using a needs assessment or pilot.
  Intentionally involve key stakeholders, field experts, and research assistants throughout program design and implementation. Select research mentors matched in skill set and availability, and consider various mentorship structures, including team‑based or tiered mentoring. Effective mentorship can inspire an “inter‑generational cascade” of researchers, contributing to programmatic sustainability [72]. Design programs flexibly, utilizing best practices for adult learning [73] by promoting interactivity [74] and experiential learning [75] while accommodating clinicians’ busy schedules. In virtual environments, prioritize building psychological safety, respect, and community. Consider a hybrid or blended approach to reach these goals [66, 75]. Conduct a thorough needs assessment to understand and prioritize LMIC participants’ needs, thereby addressing an important gap identified in Huber’s 2015 review of HRCS tools [76].
3. Prioritize equity as a targeted area for impact.
  The few programs in our sample that seemed most successful in impacting equity were those that explicitly targeted equity via program design and evaluation. Specific strategies to build equity include 1) providing targeted support to marginalized prospective participants, addressing language, time, and financial constraints throughout the program life cycle, and 2) promoting leadership and power‑sharing agreements, such as involving co‑principal investigators in multi‑site programs and establishing authorship guidelines to ensure representation of LMIC authors in publications. A significant number of publications on LMIC HRCS programs in our sample lacked LMIC authors, suggesting a critical area for improvement. To effectively measure the return on investment in health research, it is essential to disaggregate indicator data by equity categories [32]. The 2021 Research for Health Justice framework provides further guidance on operationalizing global health equity considerations in research capacity and grants programs [34].
4. Evaluate and monitor the program rigorously over time.
  Many programs lacked a stated evaluation design. Of the few articles that met criteria for MMAT [41] quality appraisal (Supplement 4), only seven had a low risk of bias. This underscores that more rigorous program evaluation is needed, consistent with Dean et al.’s scoping review of HRCS in LMICs [3]. Longitudinal evaluations are essential for capturing longer‑term impacts on equity and sustainability and those at organizational/systems levels. Evaluations should prioritize programs’ utility to LMIC(s) and utilize frameworks such as the modified RCDi to ensure actionable insights. Sufficient resources should be allocated for the evaluation of program implementation.
5. Adopt a comprehensive understanding of program sustainability.
  In HRCS in LMIC contexts, sustainability involves more than just maintaining the program itself; the ultimate aim is achieving long‑term self‑sufficiency, following the principle for any HIC partner to “work (them)self out of a job” [77]. The modified RCDi framework provides guidance for this by emphasizing leadership development, local ownership, research infrastructure, and co‑production of research across individual, organizational, and systems levels. Key considerations include ensuring ongoing access to program resources for participants, fostering research networks beyond program completion, and addressing organizational and systemic challenges from program inception. For grant‑funded programs, planning for sustainability beyond initial funding is crucial to prevent program collapse. Additionally, embedding efforts to enhance local research culture [63] contributes to system‑level sustainability.

### Limitations and future directions

Our review has several limitations. We noted a clear publication bias where authors readily report positive impacts but rarely discuss lack of impact. Most articles were missing a cohesive evaluation strategy to measure key HRCS impact domains and thus were unlikely to discuss non‑impacts. This lack of rigor in regard to evaluation, a common finding between our study and other HRCS review articles [3, 4, 26], also means that results of this review should be interpreted with caution. While our authorship team includes LMIC authors with experience in implementing virtual HRCS programs in sub‑Saharan Africa, most of the review was completed by U.S. authors. Additionally, by choosing to stop our qualitative coding after reaching theoretical saturation, we may have omitted relevant data or contextual information [39]. We endeavored to limit this by conducting a second search and comparing our completed analysis against a new group of full‑text articles meeting inclusion criteria. Our searches occurred just before the COVID‑19 pandemic. Notably absent are references to programs utilizing Zoom, now ubiquitous for online courses [78]. We encourage further study into virtual HRCS to incorporate new knowledge into the insights offered here.

## Conclusions

Virtual HRCS for LMIC clinicians is inherently an effort to improve equity within health research globally. The current literature lacks explicit measurements of equity impacts and exhibits low evaluation rigor. The modified RCDI framework (Figure 1) can guide improved program design and novel evaluation strategies to advance the HRCS field. Recommendations from this review include selecting well‑fitted program participants, intentionally designing program structure and content, conducting needs assessments or pilots, incorporating equity as a programmatic target; ensuring longitudinal program evaluation and monitoring, and utilizing a comprehensive conceptualization of program sustainability. Others may use this work to guide the next generation of virtual HRCS programs for clinicians worldwide.

## Additional Files

The additional files for this article are as follows:

Supplementary Table 1. Detrended Oscillation and Clock Parameters.

Supplementary Table 2. Characteristics of Additional Publications Meeting Inclusion Criteria (*n* = 39)^a^.

Supplementary Table 3. Publication, Program, and Evaluation Characteristics of Sample (*n* = 28 articles).

Supplementary Table 4. Mixed Methods Appraisal Tool41 Quality Appraisal of Eligible Studies (*n* = 13 articles)^a^.

Supplementary Table 5. Facilitators and Barriers to Health Research Capacity Strengthening (HRCS) Programs with Illustrative Quotes.
