# Supplementary material for: Virtual Health Research Capacity Strengthening in Low- and Middle‑Income Countries: A Systematic Integrative Review
Source: Ann Glob Health. 2025 Mar 11;91(1):14. doi: 10.5334/aogh.4543 (PMC11908432; doi:10.5334/aogh.4543)
Supplement: Supplementary Table 1. — Detrended Oscillation and Clock Parameters. [file agh-91-1-4543-s1.pdf]

Supplement 1. Systematic Search Strategy

**PubMed**
(("Capacity Building"[Mesh] AND ("Research"[Mesh]) OR "research capacity building" [TIAB] OR "research capacity strengthening"[TIAB] OR "research capacity development" [TIAB] OR "Research Personnel/education" [Mesh]) AND ((((((“Afghanistan”[Mesh] OR Afghanistan OR “Albania” [Mesh] OR Albania OR “Algeria” [Mesh] OR Algeria OR “American Samoa” [Mesh] OR “American Samoa” OR “Angola” [Mesh] OR Angola OR “Argentina” [Mesh] OR Argentina OR “Armenia” [Mesh] OR Armenia OR “Armenian SSR” OR “Armenian S.S.R.” OR “Azerbaijan” [Mesh] OR Azerbaijan OR “Azerbaijan SSR” OR “Azerbaijan S.S.R.” OR “Bangladesh” [Mesh] OR “Bangladesh” OR “Republic of Belarus” [Mesh] OR “Republic of Belarus” OR Belarus OR “Byelorussian S.S.R.” OR “Byelorussian SSR” OR “Belorussian S.S.R.” OR “Belorussian SSR” OR Byelarus OR Belorussia OR “Belize” [Mesh] OR Belize OR “Benin” [Mesh] OR “Republic of Benin” OR Dahomey OR “Bhutan” [Mesh] OR Bhutan OR “Bolivia” [Mesh] OR Bolivia OR “Bosnia and Herzegovina” [Mesh] OR “Bosnia and Herzegovina” OR Bosnia OR “Bosnia and Hercegovina” OR “Bosnia-Herzegovina” OR “Botswana” [Mesh] OR Bechuanaland OR Kalahari OR “Brazil” [Mesh] OR Brazil “Bulgaria” [Mesh] OR Bulgaria OR Burkina Faso” [Mesh] OR “Burkina Faso” OR “Upper Volta” OR “Burundi” [Mesh] OR Burundi OR “Republic of Burundi” OR Urundi OR “Cabo Verde” [Mesh] OR “Cabo Verde” OR “Republic of Cape Verde” OR “Cape Verde” OR “Cambodia” [Mesh] OR Cambodia OR “Khmer Republic” OR Kampuchea OR “Cameroon” [Mesh] OR Cameroon OR “Republic of Cameron” OR “United Republic of Cameroon” OR “Cameroons” OR “Central African Republic” [Mesh] OR “Central African Republic” OR “Chad” [Mesh] OR Chad OR “China” [Mesh] OR China OR “People’s Republic of China” OR “Mainland China” OR “Manchuria” OR Sinkiang OR “Inner Mongolia” OR “Colombia” [Mesh] OR Colombia OR “Comoros” [Mesh] OR Comoros OR “Iles Comores” OR “Comoro Islands” OR Mayotte OR “Democratic Republic of the Congo” [Mesh] OR “Democratic Republic of the Congo” OR “Congo (Kinshasa)” OR “Zaire” OR “Belgian Congo” OR “Katanga” OR “Congo” [Mesh] OR Congo OR “Republic of the Congo” OR “Congo (Brazzaville)” OR “Costa Rica” [Mesh] OR “Costa Rica” OR “Cote d'Ivoire” [Mesh] OR “Cote d’lvoire” Or “Ivory Coast” OR “Republic of Cote d’Ivoire” OR “Cuba” [Mesh] OR Cuba OR “Djibouti” [Mesh] OR Djibouti OR “Somaliland, French” OR “Republic of Djibouti” OR “French Somaliland” OR “Dominica” [Mesh] OR Dominica OR “West Indies “[Mesh] OR “Dominican Republic” [Mesh] OR “Dominican Republic” OR “Ecuador” [Mesh] OR Ecuador OR “Galapagos Islands” OR “Egypt” [Mesh] OR Egypt OR “Arab Republic of Egypt” OR “United Arab Republic” OR “El Salvador” [Mesh] OR “El Salvador” OR “Equatorial Guinea” [Mesh] OR Equatorial Guinea” OR “Republic of Equatorial Guinea” OR “Spanish Guinea” OR “Guinea, Spanish” OR “Rio Muni” OR “Eritrea” [Mesh] OR Eritrea OR “Ethiopia” [Mesh] OR Ethiopia OR “Federal Democratic Republic of Ethiopia” OR “Fiji” [Mesh] OR Fiji OR “Gabon” [Mesh] OR Gabon OR “Gabonese Republic” OR “Gambia” [Mesh] OR Gambia OR “Republic of the Gambia” OR “Georgia (Republic)” [Mesh] OR Georgia OR “Georgia SSR” OR “Republic of Georgia” OR “Georgian SSR” OR “Georgia (Western Asia)” OR “Georgian S.S.R.” OR “Ghana” [Mesh] OR Ghana OR “Republic of Ghana” OR “Gold Coast” OR “Grenada” [Mesh] OR Grenada OR “Guatemala” [Mesh] OR Guatemala OR “Guinea” [Mesh] OR Guinea OR “Guinea, French” OR “Republic of Guinea” OR “French Guinea” OR “Guinea, Republic of” OR “Guinea-Bissau” [Mesh] OR “Guinea-Bissau” OR “Republic of Guinea-Bissau” OR “Portuguese Guinea” OR “Guinea, Portuguese” OR “Guinea-Bissau, Republic of” OR “Guyana” [Mesh] OR Guyana OR “British Guiana” OR “Guiana, British” OR “Haiti” [Mesh] OR Haiti OR “Honduras” [Mesh] OR Honduras OR “India” [Mesh] OR India OR “Republic of India” OR “Indonesia” [Mesh] OR Indonesia OR “Netherlands East Indies” OR “East Indies” OR “West Irian” OR “New Guinea, Indonesian” OR “New Guinea, West” OR “Indonesian New Guinea” OR “Irian Jaya” OR “Timor” OR “Java” OR “Bali” OR “Sumatra” OR “Celebes” OR “Sulawesi” OR “Malay Archipelago” OR “Madoera” OR “Madura” OR “Iran” [Mesh] OR Iran OR “Islamic Republic of Iran” OR “Iraq” [Mesh] OR Iraq OR “Republic of Iraq” OR “Jamaica” [Mesh] OR Jamaica OR “Jordan” [Mesh] OR Jordan OR “Kazakhstan” [Mesh] OR Kazakhstan OR “Kazakh SSR” OR “Kazakh S.S.R.” OR “Kenya” [Mesh] OR Kenya OR “Republic of Kenya” OR “Democratic People's Republic of Korea” [Mesh] OR Democratic People's Republic of Korea” OR “North Korea” OR “Korea, Democratic People's Republic of” OR “Kosovo” [Mesh] OR Kosovo OR “Republic of Kosovo” OR “Kyrgyzstan” [Mesh] OR Kyrgyzstan OR Kirghizia OR “Kyrgyz Republic” OR “Kirghiz SSR” OR “Laos” [Mesh] OR Laos OR “Lebanon” [Mesh] OR Lebanon OR “Lebanese Republic” OR “Lesotho” [Mesh] OR Lesotho OR “Basutoland” OR “Kingdom of Lesotho” OR “Liberia” [Mesh] OR Liberia OR “Republic of Liberia” OR “Libya” [Mesh] OR Libya OR “Macedonia (Republic)” [Mesh] OR Macedonia OR “Former Yugoslav Republic of Macedonia” OR “Macedonia, Former Yugoslav Republic of” OR “Macedonia (Yugoslavia)” OR “The Former Yugoslav Republic of Macedonia” OR Yugoslavia OR “Madagascar” [Mesh] OR Madagascar OR “Malagasy Republic” OR “Malawi” [Mesh] OR Malawi OR “Republic of Malawi” OR Nyasaland OR “Malaysia” [Mesh] OR Malaysia OR “Federation of Malaya” OR Malaya OR “Malay Federation” OR “Malaya Federation” OR “Sabah” OR Sarawak OR “Malay Peninsula OR “Indian Ocean Islands” [Mesh] OR “Indian Ocean Islands” OR Pemba OR “Cocos (Keeling) Islands” OR “Maldives” OR “Mali” [Mesh] OR Mali OR “Republic of Mali” OR “Mauritania” [Mesh] OR Mauritania OR “Mauritius” [Mesh] OR Mauritius OR “Agalega Islands” OR “Mexico” [Mesh] OR Mexico OR “Micronesia” [Mesh] OR Micronesia OR “Johnston Island” OR Kiribati OR “Gilbert Islands” OR “Mariana Islands” OR “Marshall Islands” OR “Nauru” OR “Northern Mariana Islands” OR “Pacific Islands(Trust Territory)” OR “Tuvalu” OR “Ellice Islands” OR “Caroline Islands” OR “Micronesia, Federated States of” OR “Moldova” [Mesh] OR

Moldova OR "Moldavia" OR "Moldavian SSR" OR "Moldavian S.S.R." OR "Mongolia" [Mesh] OR Mongolia OR "Montenegro" [Mesh] OR Montenegro OR "Morocco" [Mesh] OR Morocco OR Ifni OR "Mozambique" [Mesh] OR Mozambique OR "Republic of Mozambique" OR "Portuguese East Africa" OR "Myanmar" [Mesh] OR Myanmar OR Burma OR "Namibia" [Mesh] OR Namibia OR "Southwest Africa" OR "Republic of Namibia" OR "South West Africa" "Nepal" [Mesh] OR Nepal OR "Federal Democratic Republic of Nepal" OR "Nicaragua" [Mesh] OR Nicaragua OR "Niger" [Mesh] OR Niger OR "Republic of Niger" OR "Nigeria" [Mesh] OR Nigeria OR "Federal Republic of Nigeria" OR "Pakistan" [Mesh] OR Pakistan OR "Islamic Republic of Pakistan" OR "Panama" [Mesh] OR Panama OR "Papua New Guinea" [Mesh] OR "Papua New Guinea" OR "New Guinea, Papua" OR "New Guinea, East" OR "Paraguay" [Mesh] OR "Paraguay" OR "Peru" [Mesh] OR Peru OR "Philippines" [Mesh] OR Philippines OR "Romania" [Mesh] OR Romania OR Rumania OR Roumania OR "Russia" [Mesh] OR Russia OR "Russian SFSR" OR "Russian Federation (Europe)" OR "Russian S.F.S.R." OR "Rwanda" [Mesh] OR Rwanda OR "Republic of Rwanda" OR Ruanda OR "Samoa" [Mesh] OR Samoa OR "Samoan Islands" OR "Samoa Islands" OR "Navigator Island" OR "Navigator Islands" OR "Pacific Islands "[Mesh] OR "Micronesia-Polynesia" OR "Western Samoa "[Mesh] OR "Samoa, Independent State" OR "Western Samoa" OR "Sao Tome and Principe" [Mesh] OR "Sao Tome and Principe" OR "Senegal" [Mesh] OR Senegal OR "Republic of Senegal" OR "Serbia" [Mesh] OR Serbia OR "Sierra Leone" [Mesh] OR "Sierra Leone" OR "Republic of Sierra Leone" OR "Melanesia" [Mesh] OR Melanesia OR "Norfolk Island" OR "Solomon Islands" OR "British Solomon Islands" OR "Somalia" [Mesh] OR Somalia OR "South Africa" [Mesh] OR "South Africa" OR "Union of South Africa" OR "Republic of South Africa" OR "South Sudan" [Mesh] OR "South Sudan" OR "Sri Lanka" [Mesh] OR "Sri Lanka" OR Ceylon OR "Saint Lucia" [Mesh] OR "Saint Lucia" OR "St. Lucia" OR "Saint Vincent and the Grenadines" [Mesh] OR "Saint Vincent and the Grenadines" OR "St. Vincent and the Grenadines" OR Grenadines OR "Sudan" [Mesh] OR Sudan OR "Republic of the Sudan" OR "Suriname" [Mesh] OR Suriname OR "Dutch Guiana" OR "Netherlands Guiana" OR Surinam OR "Syria" [Mesh] OR Syria OR "Tajikistan" [Mesh] OR Tajikistan OR Tadjikistan OR "Tadzhik S.S.R." OR "Tadzhik SSR" OR Tadzhikistan OR "Tanzania" [Mesh] OR Tanzania OR "United Republic of Tanzania" OR Zanzibar OR Tanganyika OR "Thailand" [Mesh] OR Thailand OR "Kingdom of Thailand" OR Siam OR "Timor-Leste" [Mesh] OR "Timor-Leste" OR "Democratic Republic of Timor-Leste" OR "East Timor" OR "Togo" [Mesh] OR Togo OR "Togolese Republic" OR "Tonga" [Mesh] OR Tonga OR "Tunisia" [Mesh] OR Tunisia OR "Turkey" [Mesh] OR Turkey OR "Turkmenistan" [Mesh] OR "Turkmenistan" OR "Turkmen SSR" OR "Turkmen S.S.R" OR "Uganda" [Mesh] OR Uganda OR "Republic of Uganda" OR "Ukraine" [Mesh] OR Ukraine OR "Uzbekistan" [Mesh] OR Uzbekistan OR "Uzbek SSR" OR "Republic of Uzbekistan" OR "Uzbek S.S.R." OR "Vanuatu" [Mesh] OR Vanuatu OR "New Hebrides" OR "Venezuela" [Mesh] OR Venezuela OR "Vietnam" [Mesh] OR Vietnam OR "Viet Nam" OR "Vietnam, Republic of" OR "North Vietnam" OR "Yemen" [Mesh] OR Yemen OR "Republic of Yemen" OR "Democratic Yemen" OR "Sanaa" OR "North Yemen" OR Aden OR "South Yemen" OR "Zambia" [Mesh] OR Zambia OR "Rhodesia, Northern" OR "Northern Rhodesia" OR "Republic of Zambia" OR "Zimbabwe" [Mesh] OR Zimbabwe OR "Zimbabwe Rhodesia" OR "Southern Rhodesia" OR "Republic of Zimbabwe" OR "Rhodesia, Southern" OR "West Bank" OR "Gaza Strip" OR "Gaza Strip (Palestine)" OR "Palestine"))))) OR ((("Developing Countries"[Mesh] OR Countries, Developing OR Country, Developing OR Developing Country OR Least Developed Countries OR Countries, Least Developed OR Country, Least Developed OR Developed Countries, Least OR Developed Country, Least OR Least Developed Country OR Less-Developed Countries OR Countries, Less-Developed OR Country, Less-Developed OR Less Developed Countries OR Less-Developed Country OR Under-Developed Nations OR Nation, Under-Developed OR Nations, Under-Developed OR Under Developed Nations OR Third-World Countries OR Countries, Third-World OR Country, Third-World OR Third World Countries OR Third-World Country OR Third-World Nations OR Nation, Third-World OR Nations, Third-World OR Third World Nations OR Third-World Nation OR Under-Developed Countries OR Countries, Under-Developed OR Country, Under-Developed OR Under Developed Countries OR Under-Developed Country OR Developing Nations OR Developing Nation OR Nations, Developing OR Less-Developed Nations OR Less Developed Nations OR Less-Developed Nation OR Nation, Less-Developed OR Nations, Less-Developed))))

Embase

capacity building'/exp AND ('research capacity building' OR 'research capacity strengthening' OR 'research capacity development' OR 'research'/exp) AND ('afghanistan'/exp OR 'afghanistan' OR 'albania'/exp OR 'albania' OR 'algeria'/exp OR 'algeria' OR 'american samoa'/exp OR 'american samoa' OR 'samoa, american' OR 'angola'/exp OR 'angola' OR 'argentina'/exp OR 'argentina' OR 'armenia'/exp OR 'armenia' OR 'armenian s.s.r.' OR 'armenian ssr' OR 'armenian soviet socialist republic' OR 'republic of armenia' OR 'azerbaijan'/exp OR 'azerbaijan' OR 'azerbaijan s.s.r.' OR 'azerbaijan ssr' OR 'azerbaijan soviet socialist republic' OR 'republic of azerbaijan' OR 'bangladesh'/exp OR 'bangladesh' OR 'east pakistan' OR 'belarus'/exp OR 'belarus' OR 'byelarus' OR 'byelorussia' OR 'byelorussian s.s.r.' OR 'byelorussian ssr' OR 'byelorussian soviet socialist republic' OR 'republic of belarus' OR 'white russia' OR 'belize'/exp OR 'belize' OR 'british honduras' OR 'benin'/exp OR 'benin' OR 'dahomey' OR 'bhutan'/exp OR 'bhutan' OR 'bolivia'/exp OR 'bolivia' OR 'bosnia and herzegovina'/exp OR 'bosnia herzegovina' OR 'bosnia and herzegovina' OR 'bosnia and herzegowina' OR 'bosnia-herzegovina' OR 'botswana'/exp OR 'botswana' OR 'bechuanaland' OR 'brazil'/exp OR 'brazil' OR 'federative republic of brazil' OR 'united states of brazil' OR 'bulgaria'/exp OR 'bulgaria' OR 'burkina faso'/exp OR 'burkina faso' OR 'upper volta' OR 'burundi'/exp OR 'burundi' OR 'cape verde'/exp OR 'cabo verde' OR 'cape verde' OR 'cape verde republic' OR 'republic of cape verde' OR 'cambodia'/exp OR 'cambodia' OR 'kampudja'

OR 'khmer republic' OR 'cameroon'/exp OR 'cameroon' OR 'cameroun' OR 'central african republic'/exp OR 'centrafrican republic' OR 'centrafrique' OR 'central african empire' OR 'central african republic' OR 'china'/exp OR 'beijing' OR 'china' OR 'chinese people`s republic' OR 'people`s republic of china' OR 'chad'/exp OR 'chad' OR 'tchad' OR 'colombia'/exp OR 'colombia' OR 'columbia' OR 'comoros'/exp OR 'comoros' OR 'comoro islands' OR 'democratic republic congo'/exp OR 'democratic republic congo' OR 'zaire' OR 'belgian congo' OR 'congo leopoldville' OR 'democratic republic of the congo' OR 'costa rica'/exp OR 'costa rica' OR 'cote d`ivoire'/exp OR 'ivory coast' OR 'cuba'/exp OR 'cuba' OR 'djibouti'/exp OR 'djibouti' OR 'afar and issa land' OR 'french sudan' OR 'dominica'/exp OR 'dominica' OR 'caribbean islands'/exp OR 'caribbean islands' OR 'indies, west' OR 'west indies' OR 'dominican republic'/exp OR 'dominican republic' OR 'ecuador'/exp OR 'ecuador' OR 'galapagos islands' OR 'egypt'/exp OR 'egypt' OR 'united arab republic' OR 'el salvador'/exp OR 'el salvador' OR 'salvador' OR 'equatorial guinea'/exp OR 'equatorial guinea' OR 'guinea, equatorial' OR 'rio muni' OR 'eritrea'/exp OR 'eritrea' OR 'ethiopia'/exp OR 'ethiopia' OR 'fiji'/exp OR 'fiji' OR 'gabon'/exp OR 'gabon' OR 'gaboon' OR 'gambia'/exp OR 'gambia' OR 'the gambia' OR 'georgia (republic)'/exp OR 'georgia (republic)' OR 'georgian s.s.r.' OR 'georgian ssr' OR 'georgian soviet socialist republic' OR 'ghana'/exp OR 'ghana' OR 'grenada'/exp OR 'grenada' OR 'guatemala'/exp OR 'guatemala' OR 'guinea'/exp OR 'guinea' OR 'guinea bissau'/exp OR 'guinea-bissau' OR 'guinea bissau' OR 'portuguese guinea' OR 'guyana'/exp OR 'guyana' OR 'guiana, british' OR 'haiti'/exp OR 'haiti' OR 'honduras'/exp OR 'honduras' OR 'india'/exp OR 'india' OR 'indian union' OR 'republic of india' OR 'union of india' OR 'indonesia'/exp OR 'indonesia' OR 'iran'/exp OR 'iran' OR 'iran (islamic republic of)' OR 'islamic republic of iran' OR 'persia' OR 'iraq'/exp OR 'iraq' OR 'jamaica'/exp OR 'jamaica' OR 'jordan'/exp OR 'jordan' OR 'transjordan' OR 'kazakhstan'/exp OR 'kazak ssr' OR 'kazakh s.s.r.' OR 'kazakh ssr' OR 'kazakh soviet socialist republic' OR 'kazakhstan' OR 'republic of kazakhstan' OR 'kenya'/exp OR 'kenya' OR 'north korea'/exp OR 'korean people`s republic' OR 'north korea' OR 'democratic people`s republic of korea' OR 'korea, democratic people`s republic of' OR 'kosovo'/exp OR 'kosovo' OR 'republic of kosovo' OR 'kyrgyzstan'/exp OR 'kirghiz s.s.r.' OR 'kirghiz ssr' OR 'kirghiz soviet socialist republic' OR 'kirghizia' OR 'kyrgyz republic' OR 'kyrgyzstan' OR 'laos'/exp OR 'laos' OR 'lao people`s democratic republic' OR 'lebanon'/exp OR 'lebanon' OR 'liberia'/exp OR 'liberia' OR 'libyan arab jamahiriya'/exp OR 'libya' OR 'libyan arab jamahiriya' OR 'libia' OR 'macedonia (republic)'/exp OR 'macedonia (republic)' OR 'macedonia, the former yugoslav republic of' OR 'yugoslavia (pre-1992)'/exp OR 'yugoslavia (pre-1992)' OR 'yugoslavia'/exp OR 'yugoslavia' OR 'madagascar'/exp OR 'madagascar' OR 'malagasy republic' OR 'malawi'/exp OR 'malawi' OR 'malaysia'/exp OR 'malaya' OR 'malaysia' OR 'north borneo' OR 'maldives'/exp OR 'maldives' OR mali OR 'republic of mali' OR 'mauritania'/exp OR 'mauritania' OR 'mauretania' OR 'mauritius'/exp OR 'mauritius' OR 'mexico'/exp OR 'mexico' OR 'united mexican states' OR 'united states of mexico' OR o OR 'federated states of micronesia'/exp OR 'federated states of micronesia' OR 'micronesia' OR 'moldova'/exp OR 'moldavia' OR 'moldavian s.s.r.' OR 'moldavian ssr' OR 'moldavian soviet socialist republic' OR 'moldova' OR 'republic of moldavia' OR 'republic of moldova' OR 'mongolia'/exp OR 'mongolia' OR 'mongolian people`s republic' OR 'outer mongolia' OR 'montenegro (republic)'/exp OR 'montenegro' OR 'montenegro (republic)' OR 'morocco'/exp OR 'morocco' OR 'mozambique'/exp OR 'mozambique' OR 'mocambique' OR 'myanmar'/exp OR 'burma' OR 'myanmar' OR 'namibia'/exp OR 'namibia' OR 'southwest africa' OR 'nepal'/exp OR 'nepal' OR 'nicaragua'/exp OR 'nicaragua' OR 'niger'/exp OR 'niger' OR 'nigeria'/exp OR 'nigeria' OR 'pakistan'/exp OR 'pakistan' OR 'panama'/exp OR 'panama' OR 'panama canal zone' OR 'papua new guinea'/exp OR 'new guinea' OR 'papua' OR 'papua new guinea' OR 'papua and new guinea' OR 'paraguay'/exp OR 'paraguay' OR 'peru'/exp OR 'peru' OR 'philippines'/exp OR 'philippines' OR 'the philippines' OR 'romania'/exp OR 'romania' OR 'rumania' OR 'ussr'/exp OR 'asiatic russia' OR 'commonwealth of independent states' OR 'russia (pre-1917)' OR 'soviet union' OR 'transcaucasia' OR 'ussr' OR 'union of socialist soviet republics' OR 'union of soviet socialist republic' OR 'union of soviet socialist republics' OR 'russian federation'/exp OR 'moscow' OR 'russia' OR 'russian federation' OR 'russian s.f.s.r.' OR 'russian sfsr' OR 'russian soviet federative socialist republic' OR 'siberia' OR 'federation, russian' OR 'rwanda'/exp OR 'rwanda' OR 'samoan islands'/exp OR 'samoan islands' OR 'samoa'/exp OR 'independent state of samoa' OR 'samoa' OR 'samoa, western' OR 'samoa, independent state' OR 'western samoa' OR 'micronesia polynesia'/exp OR 'micronesia polynesia' OR 'sao tome and principe'/exp OR 'sao tome and principe' OR 'senegal'/exp OR 'senegal' OR 'serbia'/exp OR 'serbia' OR 'sierra leone'/exp OR 'sierra leone' OR 'melanesia'/exp OR 'melanesia' OR 'somalia'/exp OR 'somalia' OR 'south africa'/exp OR 'south africa' OR 'south sudan'/exp OR 'south sudan' OR 'sri lanka'/exp OR 'sri lanka' OR 'ceylon' OR 'saint lucia'/exp OR 'saint lucia' OR 'st. lucia' OR 'saint vincent and the grenadines'/exp OR 'saint vincent and the grenadines' OR 'st. vincent and the grenadines' OR 'sudan'/exp OR 'sudan' OR 'suriname'/exp OR 'suriname' OR 'surinam' OR 'syrian arab republic'/exp OR 'syria' OR 'syrian arab republic' OR 'tajikistan'/exp OR 'republic of tajikistan' OR 'tadzhik s.s.r.' OR 'tadzhik ssr' OR 'tadzhik soviet socialist republic' OR 'tadzhikistan' OR 'tajikistan' OR 'tanzania'/exp OR 'tanzania' OR 'tanganyika' OR 'tanzania, united republic of' OR 'zanzibar' OR 'thailand'/exp OR 'thailand' OR 'siam' OR 'timor leste'/exp OR 'east timor' OR 'timor' OR 'timor-leste' OR 'togo'/exp OR 'togo' OR 'tonga'/exp OR 'tonga' OR 'tunisia'/exp OR 'tunisia' OR 'tunesia' OR 'turkey (republic)'/exp OR 'istanbul' OR 'turkey (republic)' OR 'turkish republic' OR 'turkey (country)' OR 'turkmenistan'/exp OR 'turkmen s.s.r.' OR 'turkmen ssr' OR 'turkmen soviet socialist republic' OR 'turkmenian ssr' OR 'turkmenian soviet socialist republic' OR 'turkmenistan' OR 'uganda'/exp OR 'uganda' OR 'ukraine'/exp OR 'ukraina' OR 'ukraine' OR 'ukrainian s.s.r.' OR 'ukrainian ssr' OR 'ukrainian soviet socialist republic' OR 'uzbekistan'/exp OR 'republic of uzbekistan' OR 'uzbek s.s.r.' OR 'uzbek ssr' OR 'uzbek soviet socialist republic' OR 'uzbekistan' OR 'vanuatu'/exp OR 'vanuatu' OR 'venezuela'/exp OR 'venezuela' OR 'viet nam'/exp OR 'viet nam' OR 'vietnam' OR 'yemen'/exp OR 'republic of yemen' OR 'yemen' OR 'aden' OR 'south yemen' OR

'yemen democratic republic' OR 'yemen, northern' OR 'zambia'/exp OR 'zambia' OR 'northern rhodesia' OR 'zimbabwe'/exp OR 'zimbabwe' OR 'rhodesia' OR 'southern rhodesia' OR 'palestine'/exp OR 'palestine' OR 'gaza strip palestine' OR 'gaza strip' OR 'west bank' OR 'developing country'/exp OR 'country, underdeveloped' OR 'developing area' OR 'developing countries' OR 'developing country' OR 'less-developed country' OR 'third world country' OR 'under-developed country' OR 'underdeveloped countries' OR 'underdeveloped country')
**ERIC**
MAINSUBJECT.EXACT.EXPLODE("Capacity Building") AND (MAINSUBJECT.EXACT.EXPLODE("Research") OR "research capacity building" OR "research capacity strengthening" OR "research capacity development" OR MAINSUBJECT.EXACT.EXPLODE("Researchers")) AND (MAINSUBJECT.EXACT("Developing Nations") OR ("Afghanistan" OR "Albania" OR "Algeria" OR "American Samoa" OR "Angola" OR "Argentina" OR "Armenia" OR "Armenian SSR" OR "Armenian S.S.R." OR "Azerbaijan" OR "Azerbaijan SSR" OR "Azerbaijan S.S.R." OR "Bangladesh" OR "Republic of Belarus" OR "Belarus" OR "Byelorussian S.S.R." OR "Byelorussian SSR" OR "Belorussian S.S.R." OR "Belorussian SSR" OR "Byelarus" OR "Belorussia" OR "Belize" OR "Benin" OR "Republic of Benin" OR "Dahomey" OR "Bhutan" OR "Bolivia" OR "Bosnia and Herzegovina" OR "Bosnia" OR "Bosnia and Hercegovina" OR "Bosnia-Herzegovina" OR "Botswana" OR "Bechuanaland" OR "Kalahari" OR "Brazil" OR "Bulgaria" OR "Burkina Faso" OR "Upper Volta" OR "Burundi" OR "Republic of Burundi" OR "Urundi" OR "Cabo Verde" OR "Republic of Cape Verde" OR "Cape Verde" OR "Cambodia" OR "Khmer Republic" OR "Kampuchea" OR "Cameroon" OR "Republic of Cameron" OR "United Republic of Cameroon" OR "Cameroons" OR "Central African Republic" OR "Chad" OR "China" OR "People's Republic of China" OR "Mainland China" OR "Manchuria" OR "Sinkiang" OR "Inner Mongolia" OR "Colombia" OR "Comoros" OR "Iles Comores" OR "Comoro Islands" OR "Mayotte" OR "Democratic Republic of the Congo" OR "Congo (Kinshasa)" OR "Zaire" OR "Belgian Congo" OR "Katanga" OR "Congo" OR "Republic of the Congo" OR "Congo (Brazzaville)" OR "Costa Rica" OR "Cote d'Ivoire" OR "Ivory Coast" OR "Republic of Cote d'Ivoire" OR "Cuba" OR "Djibouti" OR "Somaliland, French" OR "Republic of Djibouti" OR "French Somaliland" OR "Dominica" OR "West Indies" OR "Dominican Republic" OR "Ecuador" OR "Ecuador" OR "Galapagos Islands" OR "Egypt" OR "Arab Republic of Egypt" OR "United Arab Republic" OR "El Salvador" OR "Equatorial Guinea" OR "Republic of Equatorial Guinea" OR "Spanish Guinea" OR "Guinea, Spanish" OR "Rio Muni" OR "Eritrea" OR "Ethiopia" OR "Federal Democratic Republic of Ethiopia" OR "Fiji" OR "Gabon" OR "Gabonese Republic" OR "Gambia" OR "Republic of the Gambia" OR "Georgia (Republic)" OR "Georgia" OR "Georgia SSR" OR "Republic of Georgia" OR "Georgian SSR" OR "Georgia (Western Asia)" OR "Georgian S.S.R." OR "Ghana" OR "Republic of Ghana" OR "Gold Coast" OR "Grenada" OR "Guatemala" OR "Guinea" OR "Guinea, French" OR "Republic of Guinea" OR "French Guinea" OR "Guinea, Republic of" OR "Guinea-Bissau" OR "Republic of Guinea-Bissau" OR "Portuguese Guinea" OR "Guinea, Portuguese" OR "Guinea-Bissau, Republic of" OR "Guyana" OR "British Guiana" OR "Guiana, British" OR "Haiti" OR "Honduras" OR "India" OR "Republic of India" OR "Indonesia" OR "Netherlands East Indies" OR "East Indies" OR "West Irian" OR "New Guinea, Indonesian" OR "New Guinea, West" OR "Indonesian New Guinea" OR "Irian Jaya" OR "Timor" OR "Java" OR "Bali" OR "Sumatra" OR "Celebes" OR "Sulawesi" OR "Malay Archipelago" OR "Madoera" OR "Madura" OR "Iran" OR "Islamic Republic of Iran" OR "Iraq" OR "Republic of Iraq" OR "Jamaica" OR "Jordan" OR "Kazakhstan" OR "Kazakh SSR" OR "Kazakh S.S.R." OR "Kenya" OR "Republic of Kenya" OR "Democratic People's Republic of Korea" OR "North Korea" OR "Korea, Democratic People's Republic of" OR "Kosovo" OR "Republic of Kosovo" OR "Kyrgyzstan" OR Kirghizia OR "Kyrgyz Republic" OR "Kirghiz SSR" OR "Laos" OR "Lebanon" OR "Lebanese Republic" OR "Lesotho" OR "Lesotho" OR "Basutoland" OR "Kingdom of Lesotho" OR "Liberia" OR "Republic of Liberia" OR "Libya" OR "Macedonia (Republic)" OR "Macedonia" OR "Former Yugoslav Republic of Macedonia" OR "Macedonia, Former Yugoslav Republic of" OR "Macedonia (Yugoslavia)" OR "The Former Yugoslav Republic of Macedonia" OR "Yugoslavia" OR "Madagascar" OR "Malagasy Republic" OR "Malawi" OR "Republic of Malawi" OR "Nyasaland" OR "Malaysia" OR "Federation of Malaya" OR "Malaya" OR "Malay Federation" OR "Malaya Federation" OR "Sabah" OR "Sarawak" OR "Malay Peninsula" OR "Indian Ocean Islands" OR "Indian Ocean Islands" OR "Pemba" OR "Cocos (Keeling) Islands" OR "Maldives" OR "Mali" OR "Republic of Mali" OR "Mauritania" OR "Mauritius" OR "Agalega Islands" OR "Mexico" OR "Micronesia" OR "Johnston Island" OR "Kiribati" OR "Gilbert Islands" OR "Mariana Islands" OR "Marshall Islands" OR "Nauru" OR "Northern Mariana Islands" OR "Pacific Islands(Trust Territory)" OR "Tuvalu" OR "Ellice Islands" OR "Caroline Islands" OR "Micronesia, Federated States of" OR "Moldova" OR "Moldavia" OR "Moldavian SSR" OR "Moldavian S.S.R." OR "Mongolia" OR "Montenegro" OR "Morocco" OR "Ifni" OR "Mozambique" OR "Republic of Mozambique" OR "Portuguese East Africa" OR "Myanmar" OR "Burma" OR "Namibia" OR "Southwest Africa" OR "Republic of Namibia" OR "South West Africa" OR "Nepal" OR "Federal Democratic Republic of Nepal" OR "Nicaragua" OR "Niger" OR "Republic of Niger" OR "Nigeria" OR "Federal Republic of Nigeria" OR "Pakistan" OR "Islamic Republic of Pakistan" OR "Panama" OR "Papua New Guinea" OR "New Guinea, Papua" OR "New Guinea, East" OR "Paraguay" OR "Paraguay" OR "Peru" OR "Philippines" OR "Romania" OR "Rumania" OR "Roumania" OR "Russia" OR "Russian SFSR" OR "Russian Federation (Europe)" OR "Russian S.F.S.R." OR "Rwanda" OR "Republic of Rwanda" OR "Ruanda" OR "Samoa" OR "Samoan Islands" OR "Samoa Islands" OR "Navigator Island" OR "Navigator Islands" OR "Pacific Islands " OR "Micronesia-Polynesia" OR "Samoa, Independent State" OR "Western Samoa" OR "Sao Tome and Principe" OR "Senegal" OR "Republic of Senegal" OR "Serbia" OR "Sierra Leone" OR "Republic of Sierra Leone" OR "Melanesia" OR "Norfolk Island" OR "Solomon Islands" OR "British Solomon Islands" OR "Somalia" OR "South Africa" OR

"Union of South Africa" OR "Republic of South Africa" OR "South Sudan" OR "Sri Lanka" OR "Ceylon" OR "Saint Lucia" OR "St. Lucia" OR "Saint Vincent and the Grenadines" OR "St. Vincent and the Grenadines" OR "Grenadines" OR "Sudan" OR "Republic of the Sudan" OR "Suriname" OR "Dutch Guiana" OR "Netherlands Guiana" OR "Surinam" OR "Syria" OR "Tajikistan" OR Tadjikistan OR "Tadzhik S.S.R." OR "Tadzhik SSR" OR "Tadzhikistan" OR "Tanzania" OR "United Republic of Tanzania" OR "Zanzibar" OR "Tanganyika" OR "Thailand" OR "Kingdom of Thailand" OR "Siam" OR "Timor-Leste" OR "Timor-Leste" OR "Democratic Republic of Timor-Leste" OR "East Timor" OR "Togo" OR "Togolese Republic" OR "Tonga" OR "Tunisia" OR "Turkey" OR "Turkmenistan" OR "Turkmen SSR" OR "Turkmen S.S.R" OR "Uganda" OR "Republic of Uganda" OR "Ukraine" OR "Uzbekistan" OR "Uzbek SSR" OR "Republic of Uzbekistan" OR "Uzbek S.S.R." OR "Vanuatu" OR "New Hebrides" OR "Venezuela" OR "Vietnam" OR "Viet Nam" OR "Vietnam, Republic of" OR "North Vietnam" OR "Yemen" OR "Republic of Yemen" OR "Democratic Yemen" OR "Sanaa" OR "North Yemen" OR "Aden" OR "South Yemen" OR "Zambia" OR "Rhodesia, Northern" OR "Northern Rhodesia" OR "Republic of Zambia" OR "Zimbabwe" OR "Zimbabwe Rhodesia" OR "Southern Rhodesia" OR "Republic of Zimbabwe" OR "Rhodesia, Southern" OR "West Bank" OR "Gaza Strip" OR "Gaza Strip (Palestine)" OR "Palestine" OR "Developing Countries" OR "Countries, Developing" OR "Country, Developing" OR "Developing Country" OR "Least Developed Countries" OR "Countries, Least Developed" OR "Country, Least Developed" OR "Developed Countries, Least" OR "Developed Country, Least" OR "Least Developed Country" OR "Less-Developed Countries" OR "Countries, Less-Developed" OR "Country, Less-Developed" OR "Less Developed Countries" OR "Less-Developed Country" OR "Under-Developed Nations" OR "Nation, Under-Developed" OR "Nations, Under-Developed" OR "Under Developed Nations" OR "Third-World Countries" OR "Countries, Third-World" OR "Country, Third-World" OR "Third World Countries" OR "Third-World Country" OR "Third-World Nations" OR "Nation, Third-World" OR "Nations, Third-World" OR "Third World Nations" OR "Third-World Nation" OR "Under-Developed Countries" OR "Countries, Under-Developed" OR "Country, Under-Developed" OR "Under Developed Countries" OR "Under-Developed Country" OR "Developing Nations" OR "Developing Nation" OR "Nations, Developing" OR "Less-Developed Nations" OR "Less Developed Nations" OR "Less-Developed Nation" OR "Nation, Less-Developed" OR "Nations, Less-Developed"))
**Web of Science**
TS=("Afghanistan" OR "Albania" OR "Algeria" OR "American Samoa" OR "Angola" OR "Argentina" OR "Armenia" OR "Armenian SSR" OR "Armenian S.S.R." OR "Azerbaijan" OR "Azerbaijan SSR" OR "Azerbaijan S.S.R." OR "Bangladesh" OR "Republic of Belarus" OR "Belarus" OR "Byelorussian S.S.R." OR "Byelorussian SSR" OR "Belorussian S.S.R." OR "Belorussian SSR" OR "Byelarus" OR "Belorussia" OR "Belize" OR "Benin" OR "Republic of Benin" OR "Dahomey" OR "Bhutan" OR "Bolivia" OR "Bosnia and Herzegovina" OR "Bosnia" OR "Bosnia and Hercegovina" OR "Bosnia-Herzegovina" OR "Botswana" OR "Bechuanaland" OR "Kalahari" OR "Brazil" OR "Bulgaria" OR "Burkina Faso" OR "Upper Volta" OR "Burundi" OR "Republic of Burundi" OR "Urundi" OR "Cabo Verde" OR "Republic of Cape Verde" OR "Cape Verde" OR "Cambodia" OR "Khmer Republic" OR "Kampuchea" OR "Cameroon" OR "Republic of Cameron" OR "United Republic of Cameroon" OR "Cameroons" OR "Central African Republic" OR "Chad" OR "China" OR "People's Republic of China" OR "Mainland China" OR "Manchuria" OR "Sinkiang" OR "Inner Mongolia" OR "Colombia" OR "Comoros" OR "Iles Comores" OR "Comoro Islands" OR "Mayotte" OR "Democratic Republic of the Congo" OR "Congo (Kinshasa)" OR "Zaire" OR "Belgian Congo" OR "Katanga" OR "Congo" OR "Republic of the Congo" OR "Congo (Brazzaville)" OR "Costa Rica" OR "Cote d'Ivoire" OR "Ivory Coast" OR "Republic of Cote d'Ivoire" OR "Cuba" OR "Djibouti" OR "Somaliland, French" OR "Republic of Djibouti" OR "French Somaliland" OR "Dominica" OR "West Indies" OR "Dominican Republic" OR "Ecuador" OR "Ecuador" OR "Galapagos Islands" OR "Egypt" OR "Arab Republic of Egypt" OR "United Arab Republic" OR "El Salvador" OR "Equatorial Guinea" OR "Republic of Equatorial Guinea" OR "Spanish Guinea" OR "Guinea, Spanish" OR "Rio Muni" OR "Eritrea" OR "Ethiopia" OR "Federal Democratic Republic of Ethiopia" OR "Fiji" OR "Gabon" OR "Gabonese Republic" OR "Gambia" OR "Republic of the Gambia" OR "Georgia (Republic)" OR "Georgia" OR "Georgia SSR" OR "Republic of Georgia" OR "Georgian SSR" OR "Georgia (Western Asia)" OR "Georgian S.S.R." OR "Ghana" OR "Republic of Ghana" OR "Gold Coast" OR "Grenada" OR "Guatemala" OR "Guinea" OR "Guinea, French" OR "Republic of Guinea" OR "French Guinea" OR "Guinea, Republic of" OR "Guinea-Bissau" OR "Republic of Guinea-Bissau" OR "Portuguese Guinea" OR "Guinea, Portuguese" OR "Guinea-Bissau, Republic of" OR "Guyana" OR "British Guiana" OR "Guiana, British" OR "Haiti" OR "Honduras" OR "India" OR "Republic of India" OR "Indonesia" OR "Netherlands East Indies" OR "East Indies" OR "West Irian" OR "New Guinea, Indonesian" OR "New Guinea, West" OR "Indonesian New Guinea" OR "Irian Jaya" OR "Timor" OR "Java" OR "Bali" OR "Sumatra" OR "Celebes" OR "Sulawesi" OR "Malay Archipelago" OR "Madoera" OR "Madura" OR "Iran" OR "Islamic Republic of Iran" OR "Iraq" OR "Republic of Iraq" OR "Jamaica" OR "Jordan" OR "Kazakhstan" OR "Kazakh SSR" OR "Kazakh S.S.R." OR "Kenya" OR "Republic of Kenya" OR "Democratic People's Republic of Korea" OR "North Korea" OR "Korea, Democratic People's Republic of" OR "Kosovo" OR "Republic of Kosovo" OR "Kyrgyzstan" OR Kirghizia OR "Kyrgyz Republic" OR "Kirghiz SSR" OR "Laos" OR "Lebanon" OR "Lebanese Republic" OR "Lesotho" OR "Lesotho" OR "Basutoland" OR "Kingdom of Lesotho" OR "Liberia" OR "Republic of Liberia" OR "Libya" OR "Macedonia (Republic)" OR "Macedonia" OR "Former Yugoslav Republic of Macedonia" OR "Macedonia, Former Yugoslav Republic of" OR "Macedonia (Yugoslavia)" OR "The Former Yugoslav Republic of Macedonia" OR "Yugoslavia" OR "Madagascar" OR "Malagasy Republic" OR "Malawi" OR "Republic of Malawi" OR "Nyasaland" OR "Malaysia" OR "Federation of Malaya" OR "Malaya" OR "Malay Federation" OR "Malaya

Federation" OR "Sabah" OR "Sarawak" OR "Malay Peninsula" OR "Indian Ocean Islands" OR "Indian Ocean Islands" OR "Pemba" OR "Cocos (Keeling) Islands" OR "Maldives" OR "Mali" OR "Republic of Mali" OR "Mauritania" OR "Mauritius" OR "Agalega Islands" OR "Mexico" OR "Micronesia" OR "Johnston Island" OR "Kiribati" OR "Gilbert Islands" OR "Mariana Islands" OR "Marshall Islands" OR "Nauru" OR "Northern Mariana Islands" OR "Pacific Islands(Trust Territory)" OR "Tuvalu" OR "Ellice Islands" OR "Caroline Islands" OR "Micronesia, Federated States of" OR "Moldova" OR "Moldavia" OR "Moldavian SSR" OR "Moldavian S.S.R." OR "Mongolia" OR "Montenegro" OR "Morocco" OR "Ifni" OR "Mozambique" OR "Republic of Mozambique" OR "Portuguese East Africa" OR "Myanmar" OR "Burma" OR "Namibia" OR "Southwest Africa" OR "Republic of Namibia" OR "South West Africa" OR "Nepal" OR "Federal Democratic Republic of Nepal" OR "Nicaragua" OR "Niger" OR "Republic of Niger" OR "Nigeria" OR "Federal Republic of Nigeria" OR "Pakistan" OR "Islamic Republic of Pakistan" OR "Panama" OR "Papua New Guinea" OR "New Guinea, Papua" OR "New Guinea, East" OR "Paraguay" OR "Paraguay" OR "Peru" OR "Philippines" OR "Romania" OR "Rumania" OR "Roumania" OR "Russia" OR "Russian SFSR" OR "Russian Federation (Europe)" OR "Russian S.F.S.R." OR "Rwanda" OR "Republic of Rwanda" OR "Ruanda" OR "Samoa" OR "Samoan Islands" OR "Samoa Islands" OR "Navigator Island" OR "Navigator Islands" OR "Pacific Islands " OR "Micronesia-Polynesia" OR "Samoa, Independent State" OR "Western Samoa" OR "Sao Tome and Principe" OR "Senegal" OR "Republic of Senegal" OR "Serbia" OR "Sierra Leone" OR "Republic of Sierra Leone" OR "Melanesia" OR "Norfolk Island" OR "Solomon Islands" OR "British Solomon Islands" OR "Somalia" OR "South Africa" OR "Union of South Africa" OR "Republic of South Africa" OR "South Sudan" OR "Sri Lanka" OR "Ceylon" OR "Saint Lucia" OR "St. Lucia" OR "Saint Vincent and the Grenadines" OR "St. Vincent and the Grenadines" OR "Grenadines" OR "Sudan" OR "Republic of the Sudan" OR "Suriname" OR "Dutch Guiana" OR "Netherlands Guiana" OR "Surinam" OR "Syria" OR "Tajikistan" OR Tadjikistan OR "Tadzhik S.S.R." OR "Tadzhik SSR" OR "Tadzhikistan" OR "Tanzania" OR "United Republic of Tanzania" OR "Zanzibar" OR "Tanganyika" OR "Thailand" OR "Kingdom of Thailand" OR "Siam" OR "Timor-Leste" OR "Timor-Leste" OR "Democratic Republic of Timor-Leste" OR "East Timor" OR "Togo" OR "Togolese Republic" OR "Tonga" OR "Tunisia" OR "Turkey" OR "Turkmenistan" OR "Turkmen SSR" OR "Turkmen S.S.R" OR "Uganda" OR "Republic of Uganda" OR "Ukraine" OR "Uzbekistan" OR "Uzbek SSR" OR "Republic of Uzbekistan" OR "Uzbek S.S.R." OR "Vanuatu" OR "New Hebrides" OR "Venezuela" OR "Vietnam" OR "Viet Nam" OR "Vietnam, Republic of" OR "North Vietnam" OR "Yemen" OR "Republic of Yemen" OR "Democratic Yemen" OR "Sanaa" OR "North Yemen" OR "Aden" OR "South Yemen" OR "Zambia" OR "Rhodesia, Northern" OR "Northern Rhodesia" OR "Republic of Zambia" OR "Zimbabwe" OR "Zimbabwe Rhodesia" OR "Southern Rhodesia" OR "Republic of Zimbabwe" OR "Rhodesia, Southern" OR "West Bank" OR "Gaza Strip" OR "Gaza Strip (Palestine)" OR "Palestine" OR "Developing Countries" OR "Countries, Developing" OR "Country, Developing" OR "Developing Country" OR "Least Developed Countries" OR "Countries, Least Developed" OR "Country, Least Developed" OR "Developed Countries, Least" OR "Developed Country, Least" OR "Least Developed Country" OR "Less-Developed Countries" OR "Countries, Less-Developed" OR "Country, Less-Developed" OR "Less Developed Countries" OR "Less-Developed Country" OR "Under-Developed Nations" OR "Nation, Under-Developed" OR "Nations, Under-Developed" OR "Under Developed Nations" OR "Third-World Countries" OR "Countries, Third-World" OR "Country, Third-World" OR "Third World Countries" OR "Third-World Country" OR "Third-World Nations" OR "Nation, Third-World" OR "Nations, Third-World" OR "Third World Nations" OR "Third-World Nation" OR "Under-Developed Countries" OR "Countries, Under-Developed" OR "Country, Under-Developed" OR "Under Developed Countries" OR "Under-Developed Country" OR "Developing Nations" OR "Developing Nation" OR "Nations, Developing" OR "Less-Developed Nations" OR "Less Developed Nations" OR "Less-Developed Nation" OR "Nation, Less-Developed" OR "Nations, Less-Developed") AND TS=("Capacity Building") AND TS=("Research" OR "research capacity building" OR "research capacity strengthening" OR "research capacity development" OR "Research Personnel")
**Cochrane Library**
#1: "research capacity building":ti,ab OR "research capacity strengthening":ti,ab
OR "research capacity development":ti,ab
#2: MeSH descriptor: [Capacity Building] explode all trees
#3: MeSH descriptor: [Research Personnel] explode all trees
#4: #1 OR #2 OR #3
#5: "Afghanistan" OR "Albania" OR "Algeria" OR "American Samoa" OR "Angola" OR "Argentina" OR "Armenia" OR "Armenian SSR" OR "Armenian S.S.R." OR "Azerbaijan" OR "Azerbaijan SSR" OR "Azerbaijan S.S.R." OR "Bangladesh" OR "Republic of Belarus" OR "Belarus" OR "Byelorussian S.S.R." OR "Byelorussian SSR" OR "Belorussian S.S.R." OR "Belorussian SSR" OR "Byelarus" OR "Belorussia" OR "Belize" OR "Benin" OR "Republic of Benin" OR "Dahomey" OR "Bhutan" OR "Bolivia" OR "Bosnia and Herzegovina" OR "Bosnia" OR "Bosnia and Hercegovina" OR "Bosnia-Herzegovina" OR "Botswana" OR "Bechuanaland" OR "Kalahari" OR "Brazil" OR "Bulgaria" OR "Burkina Faso" OR "Upper Volta" OR "Burundi" OR "Republic of Burundi" OR "Urundi" OR "Cabo Verde" OR "Republic of Cape Verde" OR "Cape Verde" OR "Cambodia" OR

"Khmer Republic" OR "Kampuchea" OR "Cameroon" OR "Republic of Cameron" OR "United Republic of Cameroon" OR "Cameroons" OR "Central African Republic" OR "Chad" OR "China" OR "People's Republic of China" OR "Mainland China" OR "Manchuria" OR "Sinkiang" OR "Inner Mongolia" OR "Colombia" OR "Comoros" OR "Iles Comores" OR "Comoro Islands" OR "Mayotte" OR "Democratic Republic of the Congo" OR "Congo (Kinshasa)" OR "Zaire" OR "Belgian Congo" OR "Katanga" OR "Congo" OR "Republic of the Congo" OR "Congo (Brazzaville)" OR "Costa Rica" OR "Cote d'Ivoire" OR "Ivory Coast" OR "Republic of Cote d'Ivoire" OR "Cuba" OR "Djibouti" OR "Somaliland, French" OR "Republic of Djibouti" OR "French Somaliland" OR "Dominica" OR "West Indies" OR "Dominican Republic" OR "Ecuador" OR "Ecuador" OR "Galapagos Islands" OR "Egypt" OR "Arab Republic of Egypt" OR "United Arab Republic" OR "El Salvador" OR "Equatorial Guinea" OR "Republic of Equatorial Guinea" OR "Spanish Guinea" OR "Guinea, Spanish" OR "Rio Muni" OR "Eritrea" OR "Ethiopia" OR "Federal Democratic Republic of Ethiopia" OR "Fiji" OR "Gabon" OR "Gabonese Republic" OR "Gambia" OR "Republic of the Gambia" OR "Georgia (Republic)" OR "Georgia" OR "Georgia SSR" OR "Republic of Georgia" OR "Georgian SSR" OR "Georgia (Western Asia)" OR "Georgian S.S.R." OR "Ghana" OR "Republic of Ghana" OR "Gold Coast" OR "Grenada" OR "Guatemala" OR "Guinea" OR "Guinea, French" OR "Republic of Guinea" OR "French Guinea" OR "Guinea, Republic of" OR "Guinea-Bissau" OR "Republic of Guinea-Bissau" OR "Portuguese Guinea" OR "Guinea, Portuguese" OR "Guinea-Bissau, Republic of" OR "Guyana" OR "British Guiana" OR "Guiana, British" OR "Haiti" OR "Honduras" OR "India" OR "Republic of India" OR "Indonesia" OR "Netherlands East Indies" OR "East Indies" OR "West Irian" OR "New Guinea, Indonesian" OR "New Guinea, West" OR "Indonesian New Guinea" OR "Irian Jaya" OR "Timor" OR "Java" OR "Bali" OR "Sumatra" OR "Celebes" OR "Sulawesi" OR "Malay Archipelago" OR "Madoera" OR "Madura" OR "Iran" OR "Islamic Republic of Iran" OR "Iraq" OR "Republic of Iraq" OR "Jamaica" OR "Jordan" OR "Kazakhstan" OR "Kazakh SSR" OR "Kazakh S.S.R." OR "Kenya" OR "Republic of Kenya" OR "Democratic People's Republic of Korea" OR "North Korea" OR "Korea, Democratic People's Republic of" OR "Kosovo" OR "Republic of Kosovo" OR "Kyrgyzstan" OR Kirghizia OR "Kyrgyz Republic" OR "Kirghiz SSR" OR "Laos" OR "Lebanon" OR "Lebanese Republic" OR "Lesotho" OR "Lesotho" OR "Basutoland" OR "Kingdom of Lesotho" OR "Liberia" OR "Republic of Liberia" OR "Libya" OR "Macedonia (Republic)" OR "Macedonia" OR "Former Yugoslav Republic of Macedonia" OR "Macedonia, Former Yugoslav Republic of" OR "Macedonia (Yugoslavia)" OR "The Former Yugoslav Republic of Macedonia" OR "Yugoslavia" OR "Madagascar" OR "Malagasy Republic" OR "Malawi" OR "Republic of Malawi" OR "Nyasaland" OR "Malaysia" OR "Federation of Malaya" OR "Malaya" OR "Malay Federation" OR "Malaya Federation" OR "Sabah" OR "Sarawak" OR "Malay Peninsula" OR "Indian Ocean Islands" OR "Indian Ocean Islands" OR "Pemba" OR "Cocos (Keeling) Islands" OR "Maldives" OR "Mali" OR "Republic of Mali" OR "Mauritania" OR "Mauritius" OR "Agalega Islands" OR "Mexico" OR "Micronesia" OR "Johnston Island" OR "Kiribati" OR "Gilbert Islands" OR "Mariana Islands" OR "Marshall Islands" OR "Nauru" OR "Northern Mariana Islands" OR "Pacific Islands (Trust Territory)" OR "Tuvalu" OR "Ellice Islands" OR "Caroline Islands" OR "Micronesia, Federated States of" OR "Moldova" OR "Moldavia" OR "Moldavian SSR" OR "Moldavian S.S.R." OR "Mongolia" OR "Montenegro" OR "Morocco" OR "Ifni" OR "Mozambique" OR "Republic of Mozambique" OR "Portuguese East Africa" OR "Myanmar" OR "Burma" OR "Namibia" OR "Southwest Africa" OR "Republic of Namibia" OR "South West Africa" OR "Nepal" OR "Federal Democratic Republic of Nepal" OR "Nicaragua" OR "Niger" OR "Republic of Niger" OR "Nigeria" OR "Federal Republic of Nigeria" OR "Pakistan" OR "Islamic Republic of Pakistan" OR "Panama" OR "Papua New Guinea" OR "New Guinea, Papua" OR "New Guinea, East" OR "Paraguay" OR "Paraguay" OR "Peru" OR "Philippines" OR "Romania" OR "Rumania" OR "Roumania" OR "Russia" OR "Russian SFSR" OR "Russian Federation (Europe)" OR "Russian S.F.S.R." OR "Rwanda" OR "Republic of Rwanda" OR "Ruanda" OR "Samoa" OR "Samoan Islands" OR "Samoa Islands" OR "Navigator Island" OR "Navigator Islands" OR "Pacific Islands" OR "Micronesia-Polynesia" OR "Samoa, Independent State" OR "Western Samoa" OR "Sao Tome and Principe" OR "Senegal State OR Western Samoa OR Sao Tome and Principe OR Senegal OR "Republic of Senegal" OR "Serbia" OR "Sierra Leone" OR "Republic of Sierra Leone" OR "Melanesia" OR "Norfolk Island" OR "Solomon Islands" OR "British Solomon Islands" OR "Somalia" OR "South Africa" OR "Union of South Africa" OR "Republic of South Africa" OR "South Sudan" OR "Sri Lanka" OR "Ceylon" OR "Saint Lucia" OR "St. Lucia" OR "Saint Vincent and the Grenadines" OR "St. Vincent and the Grenadines" OR "Grenadines" OR "Sudan" OR "Republic of the Sudan" OR "Suriname" OR "Dutch Guiana" OR "Netherlands Guiana" OR "Surinam" OR "Syria" OR "Tajikistan" OR Tadjikistan OR "Tadzhik S.S.R." OR "Tadzhik SSR" OR "Tadzhikistan" OR "Tanzania" OR "United Republic of Tanzania" OR "Zanzibar" OR "Tanganyika" OR "Thailand" OR "Kingdom of Thailand" OR "Siam" OR "Timor-Leste" OR "Timor-Leste" OR "Democratic Republic of Timor-Leste" OR "East Timor" OR "Togo" OR "Togolese Republic" OR "Tonga" OR "Tunisia" OR "Turkey" OR "Turkmenistan" OR "Turkmen SSR" OR "Turkmen S.S.R" OR "Uganda" OR "Republic of Uganda" OR "Ukraine" OR "Uzbekistan" OR "Uzbek SSR" OR "Republic of Uzbekistan" OR "Uzbek S.S.R." OR "Vanuatu" OR "New Hebrides" OR "Venezuela" OR "Vietnam" OR "Viet Nam" OR "Vietnam, Republic of" OR "North Vietnam" OR "Yemen" OR "Republic of Yemen" OR "Democratic Yemen" OR "Sanaa" OR "North Yemen" OR "Aden" OR "South Yemen" OR "Zambia" OR "Rhodesia, Northern" OR "Northern Rhodesia" OR "Republic of Zambia" OR "Zimbabwe" OR "Zimbabwe Rhodesia" OR "Southern Rhodesia" OR "Republic of Zimbabwe" OR "Rhodesia, Southern" OR "West Bank" OR "Gaza Strip" OR "Gaza Strip (Palestine)" OR "Palestine" OR "Developing Countries" OR "Countries, Developing" OR "Country, Developing" OR "Developing Country" OR "Least Developed Countries" OR "Countries, Least Developed" OR "Country, Least Developed" OR "Developed Countries, Least" OR "Developed Country, Least" OR "Least Developed Country" OR "Less-Developed Countries" OR "Countries, Less-Developed" OR "Country, Less-Developed" OR "Less Developed Countries"

OR “Less-Developed Country” OR “Under-Developed Nations” OR “Nation, Under-Developed” OR “Nations, Under-Developed” OR “Under Developed Nations” OR “Third-World Countries” OR “Countries, Third-World” OR “Country, Third-World” OR “Third World Countries” OR “Third-World Country” OR “Third-World Nations” OR “Nation, Third-World” OR “Nations, Third-World” OR “Third World Nations” OR “Third-World Nation” OR “Under-Developed Countries” OR “Countries, Under-Developed” OR “Country, Under-Developed” OR “Under Developed Countries” OR “Under-Developed Country” OR “Developing Nations” OR “Developing Nation” OR “Nations, Developing” OR “Less-Developed Nations” OR “Less Developed Nations” OR “Less-Developed Nation” OR “Nation, Less-Developed” OR “Nations, Less-Developed”
#6: #4 AND #5
