# Supplementary material for: Virtual Health Research Capacity Strengthening in Low- and Middle‑Income Countries: A Systematic Integrative Review
Source: Ann Glob Health. 2025 Mar 11;91(1):14. doi: 10.5334/aogh.4543 (PMC11908432; doi:10.5334/aogh.4543)
Supplement: Supplementary Table 2. — Characteristics of Additional Publications Meeting Inclusion Criteria (n = 39)a. [file agh-91-1-4543-s2.pdf]

Supplement 2: Characteristics of Additional Publications Meeting Inclusion Criteria (n = 39)[a]

**Author / Year**	**Country(s) of LMIC Participants**	**Virtual Component(s) of HRCS Program (fully virtual or hybrid)**	**DOI / Other Publication ID**
Airhihenbuwa 2011	South Africa	Online learning-by-doing (hybrid)	10.1177/1757975911404745
Akiba 2019	Malawi, Tanzania	E-mentorship, Online discussion group(s) or forum(s) (hybrid)	10.1186/s13033-019-0327-2
Arnold 2014	Guatemala	E-mentorship, online learning-by-doing (hybrid)	10.2471/BLT.13.126516
Beane 2019	India, Pakistan, Sri Lanka	Online repository of resources (hybrid)	10.1080/16549716.2019.1587893
Bissell 2014	Cook Islands, Federated States of Micronesia, Fiji, Marshall Islands, New Caledonia, Solomon Islands, Tonga, Vanuatu	E-mentorship (hybrid)	10.5588/pha.13.0091
Breman 2004	Tanzania	Online discussion group(s) or forum(s) (hybrid)	0002-9637[b]
Dhumal 2020	India	Online course, e-mentorship, online learning-by-doing (hybrid)	10.5334/aogh.2932
Edwards 2016	China, Mexico	Online repository of resources (hybrid)	10.1186/s13012-016-0478-3
Ekeroma 2013	Cook Islands, Fiji, Samoa, Solomon Islands, Tonga, Vanuatu	E-mentorship, online discussion group(s) or forum(s) (hybrid)	23998179[c]
Ekeroma 2014	Cook Islands, Fiji, Samoa and Tonga, Solomon Islands, Vanuatu	E-mentorship, online discussion group(s) or forum(s) (hybrid)	10.1186/1472-6920-14-121
Farquhar 2011	Botswana, Kenya, Tanzania, Uganda	Online discussion group(s) or forum(s) (hybrid)	10.1016/j.idc.2011.02.005
Flint-O'Kane 2020	Kenya, Malawi, Mozambique, South Africa, The Gambia, Zimbabwe	Online course (hybrid)	10.1186/s12978-020-0876-5
Gezmu 2011	Botswana, Kenya, South Africa, Tanzania, Uganda	Online course (hybrid)	10.1002/sim.4144
Hedt-Gauthier 2017	Ghana, Mozambique, Rwanda, Tanzania, Zambia	E-mentorship (hybrid)	10.1186/s12913-017-2657-6
Lalloo 2014	South Africa	Online course (hybrid)	10.1097/ACM.0000000000000354
Lescano 2008	Peru	Online course (hybrid)	10.1371/ journal.pone.0003274
Lund 2015	Ethiopia, Ghana, Malawi, South Africa, Uganda, Zimbabwe	Online course, e-mentorship, online learning-by-doing (hybrid)	10.1017/S2045796015000281
Mathai 2019	Kenya	E-mentorship, online repository of resources (hybrid)	10.1007/s40609-018-0126-8
McGuire 2020	Lesotho	Online course, e-mentorship, online learning-by-doing (hybrid)	10.4102/phcfm.v12i1.2387

Nazer 2019	Egypt, Jordan, Oman, Saudi Arabia, Sudan	Online course, e-mentorship (hybrid)	10.21203/rs.3.rs-40533/v1
Noormahomed 2017	Mozambique	E-mentorship, online repository of resources (hybrid)	10.1080/16549716.2017.1272879
Noormahomed 2018	Mozambique	Online course (hybrid)	10.29024/aogh.14
Odiaka 2018	Ghana, Nigeria, Senegal, South Africa	Online discussion group(s) or forum(s) (hybrid)	10.1200/JGO.18.00062
Oduola 2018	Nigeria	E-mentorship (hybrid)	10.1186/s12936-018-2344-z
Okewole 2020	Ethiopia, Malawi, South Africa, Zimbabwe	Online course (hybrid)	10.5334/aogh.2759
Osanjo 2016	Kenya	Online course, online discussion group(s) or forum(s), online repository of resources (hybrid)	10.1186/s13012-016-0395-5
Phillimore 2019	Jordan, Lebanon, Palestine, Syria, Tunisia, Turkey	Online repository of resources (hybrid)	10.1080/16549716.2019.1569838
Pillai 2018	Angola, Argentina, Brazil, Cameroon, China, Costa Rica, Egypt, Ethiopia, Ghana, Kenya, Malaysia, Mexico, Namibia, Nigeria, Peru, Philippines, Russian Federation, South Africa, Tanzania, Uganda, Ukraine, Vietnam, Venezuela, Zambia, Zimbabwe	E-mentorship (hybrid)	10.1186/s12909-018-1331-y
Reddy 2002	South Africa	Online learning-by-doing (hybrid)	10.1521/aeap.14.7.92.23860
Reddy 2018	Kenya, South Africa	E-mentorship (hybrid)	10.15171/ijhpm.2018.73
Sagili 2018	India	E-mentorship (hybrid)	10.1080/16549716.2018.1445467
Schneider 2016	Afghanistan, Bangladesh, Brazil, Chile, Colombia, Ecuador, Ethiopia, Ghana, Guatemala, India, Kenya, Liberia, Malawi, Nepal, Nigeria, Pakistan, Peru, South Africa, Sri Lanka, Uganda, Zimbabwe	Online course, e-mentorship, online repository of resources, online learning-by-doing (hybrid)	10.1017/gmh.2016.24
Semrau 2018	Ethiopia, India, Nepal, Nigeria, South Africa, Uganda	E-mentorship, online learning-by-doing (hybrid)	10.1017/S2045796017000452
Sewankambo 2015	Uganda	E-mentorship (hybrid)	10.1371/journal.pmed.1001784
Stillman 2006	Brazil, China, Mexico	E-mentorship (hybrid)	10.1136/tc.2005.014753
Torondel 2019	Bangladesh, Ghana, India, Kenya, Malawi, Nepal	E-mentorship, online repository of resources (hybrid)	10.1186/s12961-019-0478-2
Tumwijukye 2013	Malawi	E-mentorship, online repository of resources (hybrid)	70962862[d]
Usher 2015	Australia, Bangladesh, Bhutan, Cambodia, China, Cook Islands, Indonesia, Laos, Nepal, Niue, Palau,	E-mentorship (hybrid)	10.1016/j.nepr.2014.03.006

	Papua New Guinea, Philippines, Samoa, Solomon Islands, Tonga		
Varadaraj 2019	India, Nepal	Online repository of resources (hybrid)	10.1093/inthealth/ihy076

[a] These 39 publications are the 33 articles from the initial search plus six second search articles containing no significant new or disconfirming information
[b] ISSN
[c] PMID
[d] CORPUS ID
Abbreviations: HRCS, health research capacity strengthening; LMIC, low- and middle-income country;
