# Supplementary material for: Virtual Health Research Capacity Strengthening in Low- and Middle‑Income Countries: A Systematic Integrative Review
Source: Ann Glob Health. 2025 Mar 11;91(1):14. doi: 10.5334/aogh.4543 (PMC11908432; doi:10.5334/aogh.4543)
Supplement: Supplementary Table 3. — Publication, Program, and Evaluation Characteristics of Sample (n = 28 articles). [file agh-91-1-4543-s3.pdf]

Supplement 3. Publication, Program, and Evaluation Characteristics of Sample (n = 28 articles)

| Publication Characteristics | | n |
|---|---|---|
| LMIC Authorship | First author | 5 |
| | Second author | 8 |
| | Middle author | 15 |
| | Last author | 11 |
| | Any first, second, or last author | 14 |
| | Any author | 20 |
| Publication Year | 2000-2005 | 1 |
| | 2006-2010 | 5 |
| | 2011-2015 | 8 |
| | 2016-2020 | 14 |
| **Program Characteristics** | | **n** |
| Type of Virtual Program | Fully virtual | 7 |
| | Hybrid (partially in-person) | 21 |
| Virtual Tools Utilized | Online course | 24 |
| | Online individual mentorship or coaching | 14 |
| | Online discussion group(s) or forum(s) | 11 |
| | Online repository of resources | 5 |
| | Online learning-by-doing | 8 |
| Focus of HRCS Program | Clinical discipline-specific research (mental health/psychiatry, occupational health, sexual and reproductive health) | 9 |
| | Disease-specific research (HIV, non-communicable diseases) | 3 |
| | Non-clinical / public health research (biomedical, epidemiology, health systems and services, operational, public health, substance abuse/addiction) | 10 |
| | Research skill (biostatistics, ethics, meta-analysis) | 8 |
| Target Population: Clinician Type | Nurses | 7 |
| | Physicians | 16 |
| | Psychologists / social workers / mental health clinicians | 6 |
| | Occupational therapists / physiotherapists | 2 |
| | Clinical pharmacists / pharmacologist | 2 |
| | Midwives | 2 |

| | | |
|---|---|---|
| | Physician assistants | 1 |
| | Clinicians / clinician-researchers (not further specified) / health professionals | 14 |
| Geographic Region of Target Populations[a] | Africa | 17 |
| | East Asia and Pacific | 2 |
| | Europe and Central Asia | 1 |
| | Latin America & the Caribbean | 10 |
| | Middle East & North Africa | 2 |
| | South Asia | 11 |
| Length of HRCS Program | <6 months | 7 |
| | 6-11 months | 1 |
| | 1-2 years | 9 |
| | 3-4 years | 4 |
| | 5+ years | 5 |
| | No program length reported | 2 |
| **Evaluation Characteristics** | | **n** |
| Evaluation Design | Mixed-methods study | 4 |
| | Non-randomized study | 2 |
| | Randomized controlled trial | 2 |
| | Qualitative study | 2 |
| | Quantitative descriptive studies | 4 |
| | No evaluation design | 14 |

[a] Geographic regions as classified by the World Bank
Abbreviations: HIV, human immunodeficiency virus; HRCS, health research capacity strengthening; LMIC, low-and middle-income country
