# Supplementary material for: Virtual Health Research Capacity Strengthening in Low- and Middle‑Income Countries: A Systematic Integrative Review
Source: Ann Glob Health. 2025 Mar 11;91(1):14. doi: 10.5334/aogh.4543 (PMC11908432; doi:10.5334/aogh.4543)
Supplement: Supplementary Table 4. — Mixed Methods Appraisal Tool41 Quality Appraisal of Eligible Studies (n = 13 articles)a. [file agh-91-1-4543-s4.pdf]

Supplement 4. Mixed Methods Appraisal Tool[41] Quality Appraisal of Eligible Studies (n = 13 articles)[a]

Study Type	MMAT Questions					
**Qualitative Studies** Citation (Evaluation sample size)	1.1 Is the qualitative approach appropriate to answer the research question?	1.2 Are the qualitative data collection methods adequate to address the research question?	1.3 Are the findings adequately derived from the data?	1.4 Is the interpretation of results sufficiently substantiated by data?	1.5 Is there coherence between qualitative data sources, collection, analysis and interpretation?	**Summary of evaluation quality based on MMAT results and sample size / Reviewer's determination of high (H) versus low (L) overall risk of bias**
De Silva 2019 (5)	Yes	No	No	No	No	Qualitative design not well-elaborated, lack of rigorous analysis methodology, small sample size / H
Okewole 2020 (15)[b]	Yes	Yes	Yes	Yes	Yes	Qualitative design well-elaborated, code book presented, small sample size / L
**Randomized Controlled Trials** Citation (Evaluation sample size)	2.1. Is randomization appropriately performed?	2.2. Are the groups comparable at baseline?	2.3. Are there complete outcome data?	2.4. Are outcome assessors blinded to the intervention provided?	2.5 Did the participants adhere to the assigned intervention?	**Summary of evaluation quality based on MMAT results and sample size / Reviewer's determination of high (H) versus low (L) overall risk of bias**
Aggarwal 2011 (58)	Yes	Yes	Yes	Can't tell	Yes	Blinding not mentioned, no other concerns on evaluation quality, moderate sample size / L
Barchi 2013 (38)	Can't tell	Yes	Yes	Yes	Yes	Limited randomization details, some response bias risk (53% total response rate, 83% in virtual arm), no other evaluation quality concerns, moderate sample size / L

**Non-Randomized Studies** Citation	3.1. Are the participants representative of the target population?	3.2. Are measurements appropriate regarding both the outcome and	3.3. Are there complete outcome data?	3.4. Are the confounders accounted for in the design and analysis?	3.5. During the study period, is the intervention administered (or	**Summary of evaluation quality based on MMAT results and sample size / Reviewer's determination of high (H) versus low (L) overall risk of bias**

(Evaluation sample size)		intervention (or exposure)?			exposure occurred) as intended?	
Abawi 2016 (175)	Yes	Yes	Yes	Can't tell	Yes	Evaluation response rate 80%, low concern for hidden confounders, well designed, large sample size / L
Dodani 2008 (18)	Can't tell	Yes	No	Can't tell	Yes	Incomplete data (43% response rate) increasing risk of response bias, no discussion of confounders, duplicate near-identical publication / H
**Quantitative Descriptive Studies** Citation (Evaluation sample size)	4.1 Is the sampling strategy relevant to address the research question?	4.2 Is the sample representative of the target population?	4.3 Are the measurements appropriate?	4.4 Is the risk of nonresponse bias low?	4.5 Is statistical analysis appropriate to answer the research question?	**Summary of evaluation quality based on MMAT results and sample size / Reviewer's determination of high (H) versus low (L) overall risk of bias**
Decroo 2018 (6)	Yes	No	Yes	Yes	Yes	Sample not representative of larger target population: all participants had prior research experience and were associated with MSF, small sample size / H
Dodani 2012 (33)	Yes	Yes	Yes	Yes	Yes	No explanation of participant randomization, errors in statistical reporting, high concern for response bias, duplicate near-identical publication / H
CORDIS 2015 (NR)	Can't tell	Can't tell	Yes	Can't tell	No	Unclear risk of nonresponse bias or representativeness, no total sample size reported, statistical approach not discussed / H
Mayor 2019 (14)	Yes	Yes	Yes	Yes	Yes	Clear description of target population with appropriately selected sample, low risk of nonresponse bias, small to moderate sample size / L

**Mixed Methods Studies**<br>Citation (Evaluation sample size)	5.1. Is there an adequate rationale for using a mixed methods design to address the research question?	5.2. Are the different components of the study effectively integrated to answer the research question?	5.3. Are the outputs of the integration of qualitative and quantitative components adequately interpreted?	5.4. Are divergences and inconsistencies between quantitative and qualitative results adequately addressed?	5.5. Do the different components of the study adhere to the quality criteria of each tradition of the methods involved?	**Summary of evaluation quality based on MMAT results and sample size / Reviewer's determination of high (H) versus low (L) overall risk of bias**
Byrnes 2019 (53)	Yes	Yes	Can't tell	Yes	Can't tell	Limited detail on both quantitative and qualitative study methods, no data on response rate, moderate sample size / H
McGuire 2020 (20)[b]	Yes	Yes	No	Can't tell	Yes	Gives interpretation of both quantitative and qualitative findings although not well integrated; no clear divergences between results, small to moderate sample size / L
Protsiv 2016 (18)	No	Yes	Yes	Can't tell	Yes	No explicit discussion on why mixed methods were selected, quantitative data limited but integrated well, small to moderate sample size / L

[a] Quality appraisal was conducted on articles which met the two MMAT screening questions: 'Are there clear research questions?' and 'Does the collected data allow for addressing the research questions?'
[b] Publications included from updated (March 2021) literature search which contained significant new information
Abbreviations: H, high; L, low; MSF, *Médecins Sans Frontières* (Doctors without Borders); MMAT, mixed methods appraisal tool; NR, not reported
