# Supplementary material for: Virtual Health Research Capacity Strengthening in Low- and Middle‑Income Countries: A Systematic Integrative Review
Source: Ann Glob Health. 2025 Mar 11;91(1):14. doi: 10.5334/aogh.4543 (PMC11908432; doi:10.5334/aogh.4543)
Supplement: Supplementary Table 5. — Facilitators and Barriers to Health Research Capacity Strengthening (HRCS) Programs with Illustrative Quotes. [file agh-91-1-4543-s5.pdf]

Supplement 5. Facilitators and Barriers to Health Research Capacity Strengthening Programs with Illustrative Quotes

	Topic area	Themes	Illustrative Quotes
**Virtual-specific facilitators and barriers**	E-platform	A variety of strategies for facilitating online course development and delivery were found, including selecting a familiar and/or user-friendly platform, having back-up plans for IT failure, and ensuring facilitators/lecturers have prior experience in online course delivery	"The open-source Moodle was chosen, as this was already in use in some universities and easy to install."[47]<br><br>"The facilitators of each module have experience in giving online lectures and presentations and in conducting conferences."[42]
		Barriers to e-learning included platforms that were not "user-friendly," and firewall/security problems with accessing the platforms	"The trainees found it difficult to follow the modules and exercises. This might be partly due to shortcomings of the Moodle platform interface design."[55]
	Internet access	Programs and participants employed various strategies to facilitate access, such as keeping file-sizes small and participants working mostly offline until internet was available	"Files are kept small because of internet access issues. Many of the students access the Internet from internet cafes so ease of downloading materials is very important to them."[61]
		Programs ensured participants had adequate hardware, software and/or internet connection prior to program start	"Before attending (...), each participant completed a short course about on-line learning methods; this also helped confirm that they had adequate computer hardware and internet bandwidth."[9]
		Poor internet access and/or quality, lack of access to virtual interfacing equipment, and other resource restrictions prevented participation in some programs	"We experienced challenges related to limitations in infrastructure and equipment, particularly new computer hardware and software, reliable access to the internet and communication lines, work and meeting space, and transportation issues."[56]

	Topic area	Themes	Illustrative Quotes
	Program delivery / design strategies	Some programs highlighted the benefits of scheduled, synchronous sessions, while others preferred flexible, self-directed, and asynchronous approaches, especially given time zone challenges	“The other form of flexibility – the time when the learning happened – was introduced by the self-directed part of the course (…) Combining the learning activities with these various flexible elements, as well as adding on-site support for learners, gave students at three different universities the ability to fully participate.”[60]<br><br>“Ten-hour differences in time zones, different work schedules, problems with equipment, and power outages were technical problems that made multiple members’ Skype calls impossible.”[66]
		Interactive sessions via small group work or live video sessions, the use of practical application, and "learning-by-doing" were key facilitators (or barriers when absent)	“Some students wished that the practical exercises allowed them to use their own real-world problems (…) 'I think it would have been even more useful if I had been made to apply what I had learned on a daily basis to a real example of my own. The only way one truly learns is to do.”[60]
		Course elements can be overly time-intensive and become a barrier to participation to both learners and mentors/faculty alike	“This closed online learning forum was (…) developed as a moderated forum [and] required one member (either a middle level or senior researcher) to spend 2 hours a week initially to moderate the discussions. This posed a barrier, as most senior researchers were unable to commit time to facilitate the discussions on forum”[62]
	Language considerations	Some programs used creative strategies to facilitate online communication despite language differences	“Network members were cognizant of potential language barriers. Among seven different languages across the COEs [Centers of Excellence], English was a common language, but communication was still challenging for some investigators. Staff strove to minimize the use of acronyms. Procedures were implemented to standardize messages and make it easier for everyone to communicate. Examples (…) included creating an email template for communications that noted urgency and date for response; copying additional COE staff on all COE PI [principle investigator] communications; and keeping emails short and concise by putting additional information (…) in an email attachment”[45]

	Topic area	Themes	Illustrative Quotes
		Language barriers were noted in several programs	"Another barrier (…) is language. Research is taught and published predominantly in English, the lingua franca of science. Awardees from countries where most university-level students are taught in English (e.g., India, South Africa, Nigeria) do not face the same language barriers as those trained in Spanish, Portuguese, or French."[59]
	Hybrid approach	Combing face-to-face and online components can build a sense of trust and safety; some programs reported that using in-country followed by distance e-mentorship facilitated overall effectiveness	"While a strength of this study is the sustained mentoring of participants, there are also limitations to mentoring at a distance (…) [This] has led to a recommendation to combine both in-country and distance mentoring as a more effective method."[67]
	Characteristics of virtual HRCS	The ease of adapting, evaluating, monitoring, and sharing online content were highlighted as facilitators	"On-line programs, which offer maximal flexibility in use and require relatively few resources once established, have the added advantage of accessibility for large numbers of people."[44]
		Some programs noted difficulty with measuring the degree to which participants were learning or benefiting from online content	"We do not know how many of the members actually follow and benefit from the activities and discussions (….) there is no way of knowing whether membership in the group is actually benefiting everybody."[64]
		While several articles discussed low recurring costs of virtual HRCS programs as a potential facilitator to their cost-effectiveness and scalability, few programs measured and reported on costs or scale-up efforts.	Although preparation of on-line training material may require a high initial financial investment, the recurring costs (…) are generally lower, because faculty and student travel costs are eliminated, and faculty time required for delivering didactic course material, though not for interactive sessions, is reduced."[9]
**General facilitators and barriers**	Design informed by needs assessment	A needs assessment can focus program efforts and facilitate the intentional design and delivery of course components, as there are different design considerations for online versus in-person courses	"A preliminary visit (…) [and] personal interviews conducted in June 2016 (…) showed that the hospital staff lacked previous exposure to research, and training resources were adapted to provide the basic fundamentals of health research. (…) The eLearning Platform was designed to consider the trainees' professional background [and] their role in the community or in their institutions."[55]
		Poor design, structure and/or course content were named as barriers in a few programs	"In the initial years of the training program, attempts at curriculum design lacked information regarding the appropriate curriculum elements (i.e., content and skills) that would ensure post-training success of participants."[63]

	Topic area	Themes	Illustrative Quotes
	Participant characteristics	Participants selection to ensure inclusion of those the right point in their research career and are highly motivated was discussed as a facilitator	"Selecting the right set of professionals who are interested and keen in taking on research as their career is important."[49]
		Some programs reported a lack of uniform prerequisite skills amongst trainees as a barrier	"Our training program attracted applicants from different specialties, at different stages in their careers, and with different learning styles, which presented challenges in developing a pedagogy that is responsive to the needs of all trainees."[63]
	Program personnel characteristics	Intentional involvement of specific stakeholders, mentors, peer mentors, speakers, field experts, research assistants, graduates and other students can facilitate HRCS programs	"The caliber and number of mentors and the value of the close relationships with the mentors were mentioned as well. Having close contact with international speakers and renowned researchers was also appreciated."[54]<br><br>"Graduate students (…) volunteered to give statistical support to move manuscripts from thesis to publication. The UON [University of Nairobi] trainee would send their de-identified datasets (…) to the UW [University of Washington] (…) graduate students, [who] were grateful to be added as co-authors."[66]
		Matching the availability and appropriate expertise of mentors and mentees was a barrier for some programs	"Matching students' topics and experts' areas of interest was a challenge."[43]
	Program accessibility	Various strategies were used to increase accessibility: making the training free, supporting research project and dissemination costs, and ensuring participants have the necessary know-how, technology and/or physical resources available	"Modest financial support was provided to trainees to support their research, attendance at program events, and the dissemination of their research findings for at least one international conference."[46]
	Research environment	An open, positive, and respectful atmosphere within the program is an important facilitator	"Central to effective mentoring is the establishment of respectful relationships (Redman-MacLaren et al., 2012) that do not discriminate against individual participant's depth of research experience and value both the individual and collective wisdom of the group. Findings from this study report participants established respectful relationships with each other and with workshop facilitators."[67]
		A challenging external climate, such as political tensions, unfavorable administrative regulations, lack	"The core capacity-building team of SHARE faced challenges resulting from cross-border tensions that made it hard for researchers from certain regions to participate (…) Different sets of

	Topic area	Themes	Illustrative Quotes
		of research culture or infrastructure, posed barriers to some programs	administrative regulations across the institutes in the region led to complications and delays in starting or sustaining certain capacity-building activities."[62]<br><br>"For example, the region witnessed frequent turnover of top officials at universities with whom we had established relationships, and hence, continuity of collaborative efforts was disturbed. In particular, academia underwent profound changes in Egypt after the Arab Spring, whereby many top officials with ties to the Mubarak regime were asked to resign. Also, political tensions and continuous demonstrations led to interruptions in many trainee activities."[63]
Abbreviations: IT, information technology; HRCS, health research capacity strengthening; LMIC, low and middle-income country			
